# Camphor-10-Sulfonamide Amino Acid Esters: Synthesis, Antiviral Evaluation, and Molecular Docking Insights

**DOI:** 10.3390/ijms27020616

**Published:** 2026-01-07

**Authors:** Krasimira Dikova, Neli Vilhelmova-Ilieva, Emilio Mateev, Zhanina Petkova

**Affiliations:** 1 Laboratory Organic Synthesis and Stereochemistry, Institute of Organic Chemistry with Centre of Phytochemistry, Bulgarian Academy of Sciences, 1113 Sofia, Bulgaria; krasimira.dikova@orgchm.bas.bg; 2 Centre of Competence “Sustainable Utilization of Bio-Resources and Waste of Medicinal and Aromatic Plants for Innovative Bioactive Products” (BIORESOURCES BG), 1000 Sofia, Bulgaria; 3 The Stephan Angeloff Institute of Microbiology, Bulgarian Academy of Sciences, Acad. G. Bonchev Str. 26, 1113 Sofia, Bulgaria; 4 Department of Pharmaceutical Chemistry, Faculty of Pharmacy, Medical University—Sofia, 1000 Sofia, Bulgaria; e.mateev@pharmfac.mu-sofia.bg

**Keywords:** camphor-based sulfonamides, feasible synthesis, antiviral activity, virucidal activity, viral adsorption, HSV-1, human coronavirus, FCV, molecular docking

## Abstract

The ongoing emergence of antiviral drug resistance underscores the critical need for new broad-spectrum antiviral agents. Sulfonamides and their derivatives have emerged as promising candidates for the development of new antiviral therapeutics. In this study, a series of camphor-10-sulfonamide derivatives was synthesized through a feasible and sustainable synthetic approach starting from naturally available precursors and evaluated for antiviral properties. Their activity was examined against three structurally distinct viruses—herpes simplex virus type 1 (HSV-1), human coronavirus (HCoV-OC43), and feline calicivirus (FCV)—representing both DNA and RNA, enveloped and non-enveloped types. The compounds were examined for their effects on viral replication, the stage of viral adsorption to the cell, and extracellular virions. The weakest cytotoxicity and the most pronounced activity of all the tested substances was demonstrated by the tryptophan derivative **7a**. A time-dependent inhibition of the stage of adsorption of HCoV-OC43 (Δlg = 2.0 at 120 min) and FCV (Δlg = 1.75 at 60 min) to susceptible cells was established, as well as virucidal activity on the three types of virions tested, with the most pronounced effect at 120 min—for HSV-1 (Δlg = 2.75) and Δlg = 2.0 for HCoV-OC43 and FCV. Molecular docking studies performed using Glide (Schrödinger) provided insights into the active conformations of the most effective ligands and predicted possible interactions with relevant viral targets, supporting their potential as lead structures for further therapeutic development.

## 1. Introduction

Viruses circulating in the human population are responsible for approximately one-third of all deaths caused by infectious agents [1]. Since the discovery of the first antiviral drug, iododeoxyuridine (IDU), in 1959 [2], only a limited number of therapeutic agents have been approved for clinical use. The diverse replication strategies of different viruses make it difficult to develop a broad-spectrum antiviral therapeutic. Another obstacle to the development of effective therapeutics is the ability of viruses to rapidly select mutants that are resistant to the applied antiviral drugs. Since viral replication occurs inside the host cell, the therapeutics developed often also affect the host itself, which reduces their clinical effectiveness [3]. These limitations highlight the urgent need for new antiviral strategies that minimize host toxicity and the risk of resistance development [4]. Recently, the focus for the discovery of new antiviral agents has been directed towards natural products. They have significantly lower cytotoxicity compared to synthetic products, cause fewer side effects in the host, have better bioavailability, possess broader antiviral activity, and are less likely to induce the formation of resistant mutants.

Sulfonamides and their derivatives were the first antibacterial agents [5], known for being chemically stable and possessing good bioavailability [6]. Subsequently, many other biological activities have been proven, including antihypertensive, hypoglycemic, diuretic, antiepileptic, anti-inflammatory, antitumor, antiprotozoal, and antifungal activities, as well as activity in the treatment of neurodegenerative diseases [7,8,9,10,11,12,13,14,15,16]. Notably, sulfonamide-based derivatives have demonstrated inhibitory activity against several viruses, such as adenoviruses, coxsackievirus, enteroviruses, human parainfluenza viruses, Ebola virus, Marburg virus, SARS-CoV-2, HIV, etc. [17,18,19]. For instance, a morpholine-substituted sulfonamide showed three-fold higher activity against avian paramyxovirus serotype-1 (APMV-1) compared to ribavirin [20], while another sulfonamide analog demonstrated EC_50_ comparable to the EC_50_ of the reference drugs (ganciclovir and cidofovir) against the replication of cytomegalovirus strain AD169 [21]. Furthermore, a cyclic sulfonamide derivative showed antiviral activity against SARS-CoV-2 [22].

Camphor, a naturally abundant and affordable monoterpenoid widely distributed in plants, serves as an excellent chiral building block for drug design. Camphor derivatives have demonstrated diverse biological activities, such as antioxidant, analgesic, anticancer, antimicrobial, and antiviral activities [23]. It has been experimentally shown that a series of camphor derivatives, (*S*)-(+)-camphor-10-sulfonamides with different substituents on the nitrogen atom, possess antiviral activity against filoviruses (Ebola and Marburg). This effect was studied using a pseudovirus system based on vesicular stomatitis virus (VSV) [24].

In the present study, the antiviral activities of six camphor-based sulfonamides were investigated. They were designed as feasible and synthetically accessible molecules derived from natural products. The synthetic approach integrates low-cost, renewable chiral precursors, consistent with the principles of sustainable and practical medicinal chemistry [25,26]. Such an approach enables the efficient generation of small, structurally diverse compound libraries suitable for biological screening.

The antiviral activity of the synthesized compounds was evaluated against three structurally different viruses: herpes simplex virus type 1, (DNA, enveloped virus); human coronavirus (RNA, enveloped virus) and feline calicivirus (RNA, non-enveloped virus). The structural differences between the three viruses are the reason why they were chosen as models in the present study. Molecular docking studies using Glide XP (Schrödinger), IFD, and MM/GBSA rescoring were performed on selected compounds to generate hypothetical binding poses at viral/host targets relevant to adsorption/virucidal activity. These computational predictions provide preliminary structural hypotheses to contextualize experimental data.

## 2. Results and Discussion

### 2.1. Synthesis

**Scheme 1:**
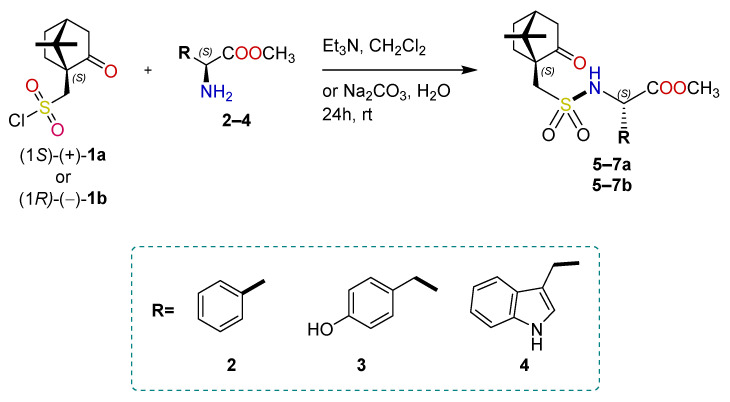
Synthesis of camphor-10-sulfonamide derivatives (**5a**,**b**–**7a**,**b**) from (*R*)- or (*S*)-camphorsulfonyl chloride (**1a** or **1b**) and amino acid methyl esters (**2**–**4**) under mild conditions (Et_3_N, CH_2_Cl_2_, or Na_2_CO_3_, H_2_O). Atom colors are used consistently throughout the manuscript to match the color coding applied in the molecular docking figures.

The synthesis of camphor-10-sulfonamide derivatives (**5a**,**b**–**7a**,**b**) was achieved through the reaction between (*R*)- or (*S*)-camphorsulfonyl chloride (**1a** or **1b**) and amino acid methyl esters (**2**–**4**) in the presence of a base under mild conditions (Scheme 1). Initially, our synthetic approach focused on the use of amino acids as nucleophiles for the preparation of camphor-based derivatives. Several literature protocols were examined and further modified [27,28,29,30]; however, under our experimental conditions, these approaches resulted in very low yields and non-isolable material with extremely low solubility, which prevented both the reliable NMR characterization of the obtained products and biological evaluation. Although the formation of the desired camphor-10-sulfonamide derivatives was likely, the available material was insufficient for further analysis. Therefore, the synthetic strategy was redirected toward the use of amino acid methyl esters, affording soluble and isolable compounds suitable for further studies. From a conceptual standpoint, this work represents a feasible and sustainable synthesis approach based on inexpensive, naturally derived chiral building blocks such as camphor and amino acid derivatives [31,32].

This method facilitates the preparation of diverse and affordable antiviral scaffolds. Despite the optimization of the reaction conditions, the isolated yields remained moderate to low (10–73%). This variation in yield reflects the steric and electronic influence of the amino acid side chains. Additional changes in the solvent or base did not improve the yields. In particular, reactions involving tyrosine and tryptophan derivatives occasionally led to the formation of disubstituted by-products, likely due to side reactions involving the nucleophilic groups of their aromatic residues. Depending on the amino ester used, the reactions were carried out either in CH_2_Cl_2_ with Et_3_N or in aqueous medium with Na_2_CO_3_, affording the desired sulfonamides in moderate yields. The amino acid methyl esters of phenylglycine, tyrosine, and tryptophan were used as nucleophiles, providing camphor-10-sulfonamides with diverse side chains. The structures of all synthesized compounds were confirmed by NMR and HRMS analyses. Melting points, specific optical rotation values, and full spectral data are provided in the Supporting Information.

The synthesized compounds were evaluated for cytotoxicity and antiviral activity against three model viruses representing different structural types, while molecular docking studies were performed to investigate their potential interactions with viral targets.

### 2.2. Determination of Cytotoxicity

Before conducting the antiviral experiments, the cytotoxicity of the tested substances was determined. The three cell lines (MDBK, VeroE6, and CRFK) were also employed for the subsequent antiviral assays. The aim was to establish the non-toxic concentration range of the tested products and to perform the antiviral assays under these conditions. Of the three cell lines studied, the cytotoxicity of the tested substances against the MDBK cell line was the weakest, the cytotoxicity observed against CRFK cells was slightly higher, and against VeroE6, the cytotoxicity was significantly higher. The results obtained are best explained by the need to determine cytotoxicity in MDBK and CRFK at 48 h of treatment, and in VeroE6 at 120 h. These hours are determined by the period during which the virus gives the most pronounced cytopathic effect. The antiviral experiments were recorded at these time intervals, and, accordingly, the influence of the cytotoxicity of the tested substances was determined at the same time intervals. In this way, the toxic effect of the tested product was excluded at the specified interval for determining its antiviral effect. The cytotoxic concentrations, which reduced the viability of the cell monolayer by 50% (CC_50_), were determined to establish whether the given substance had a selective index (SI) for the inhibition of the replication of a given viral strain. The maximum tolerated concentrations (MTC) were also determined, representing the point at which no change in the cell monolayer was observed. Experiments were conducted with the MTC to monitor the effect of this concentration on extracellular virions and their stage of adsorption to sensitive cells (Table 1).

**Figure 1:**
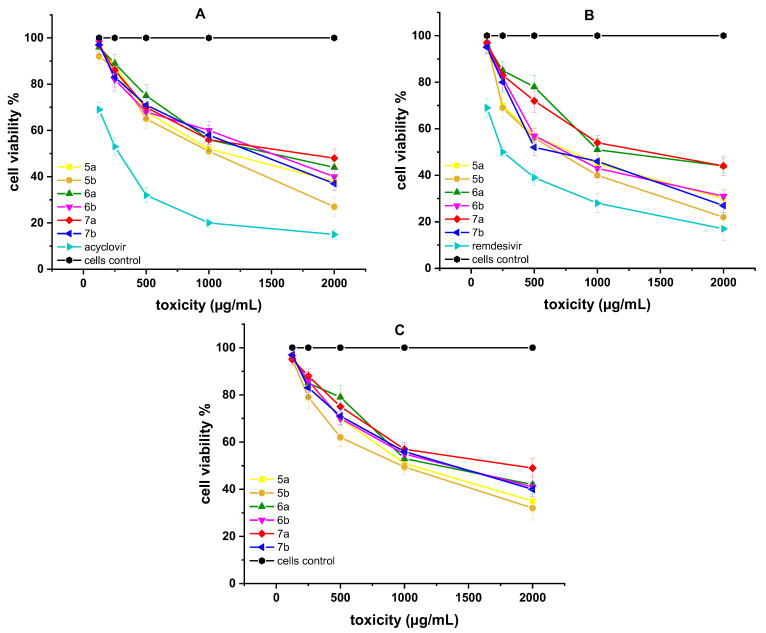
Cytotoxicity of camphor-10-sulfonamide derivatives: (**A**) cytotoxicity determined against the MDBK cell line; (**B**) cytotoxicity determined against the VeroE6 cell line; (**C**) cytotoxicity on CRFK cells. The results are presented as means ± SD. Each experiment was repeated 4 times (*n* = 4).

Among all the tested substances, **7a** exhibited the lowest cytotoxicity compared to the third tested cell line. In the MDBC and CRFK cell lines, the next in cytotoxicity, with similar CC_50_ values, are **6a** and **7b**, followed by **6b**. The pair **5a**, **5b** demonstrated higher cytotoxicity, with the highest cytotoxicity observed in **5b** (Table 1). In the VeroE6 cell line, the same dependence of toxicity was maintained, but with significantly more toxic values (Figure 1 and Table 1).

### 2.3. Antiviral Activity

#### 2.3.1. Effect on the Viral Replication Cycle

When determining the effect on the viral replication cycle in the three tested viral strains, it was found that only **7a** showed any activity. Against HSV-1 replication, it demonstrated a 50% inhibition of viral replication (IC_50_) of 1710.5 µg/mL, from which an SI = 1.11 was determined. Weak activity was also noted for HCoV-OC43 with an IC_50_ = 1117.5 µg/mL (SI = 1.16). Of the other tested substances, none showed 50% inhibition of the viral replication cycle (the percentage was lower); therefore, the SI of the substances could not be determined.

#### 2.3.2. Inhibition of the Stage of Viral Adsorption to Susceptible Cells

**Figure 2:**
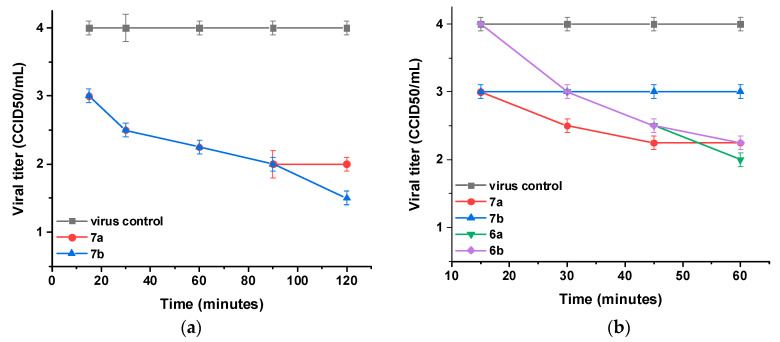
Inhibition of the viral adsorption stage: (**a**) effect on the adsorption of HCoV-OC43 to VeroE6 cells; (**b**) effect on the adsorption of FCV to CRFK cells. Each experiment was repeated 4 times (*n* = 4).

When monitoring the influence of the studied substances on the stage of viral adsorption, it was found that none of them significantly affected the process of the adsorption of HSV-1 to MDBK cells at any of the studied time intervals. In HCoV-OC43, a significant influence on adsorption to VeroE6 cells was demonstrated by **7a** and **7b**. A time/effect dependence was observed, and with increasing exposure time, the observed effect also increased. At 120 min of exposure, a decrease in the viral titer was observed with Δlg = 2.0, and with **7b**, the effect was slightly more pronounced with Δlg = 2.5. Compared to the adsorption of FCV to CRFK cells, **7a** showed a slightly weaker effect of Δlg = 1.75, and the influence of **7b** was significantly weaker (Δlg = 1.0). The **6a** and **6b** pair also showed an inhibitory effect on FCV adsorption. The activity was slightly more pronounced for **6a** (Δlg = 2.0) compared to **6b** (Δlg = 1.75). Compounds **5a** and **5b** did not show a significant inhibitory effect on the adsorption stage of any of the tested viral strains. (Figure 2, Table 2).

#### 2.3.3. Effect on Extracellular Virions

**Figure 3:**
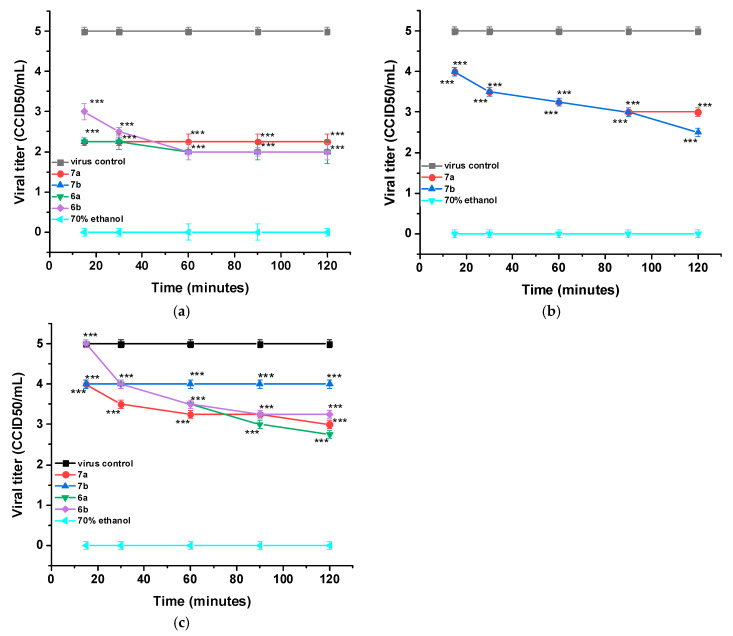
Inhibition of extracellular virions: (**a**) effect on HSV-1 virions; (**b**) effect on HCoV-OC43 virions; (**c**) effect on FCV virions. *** *p* < 0.001, when comparing the value of each tested substance with the value of 70% ethanol for the corresponding time interval, Student’s *t*-test. Each experiment was repeated 4 times (*n* = 4).

The next stage of our study was to monitor the effect of the tested substances on the viability of the extracellular virions of the three viruses involved in the research. The most distinct activity against HSV-1 virions was shown by the isomer pairs **6a**/**6b** and **7a**/**7b**, with **6a**, **6b** and **7b** showing a certain increase in activity with time and at/and after 60 min of exposure, all three substances showed a decrease in the viral titer with Δlg = 3.0. With **7a**, the effect remained the same at all studied time intervals (Δlg = 2.75) (Figure 3a, Table 3).

Only the isomers **7a** and **7b** exhibited virucidal activity against HCoV-OC43 virions, in which the effect was weak up to 30 min after exposure and increased at/and after 60 min, reaching values of Δlg = 2.0 (**7a**) and Δlg = 2.5 (**7b**) at 120 min (Figure 3b, Table 3). Against FCV virions, only **6a**, **6b**, and **7a** demonstrated activity, with no activity observed at 15 min for **6a** and **6b**, and weak inhibition for **7a**. At 30 min, all three substances showed weak activity. With increasing exposure time, the effect also increased, being most pronounced for **6a** (Δlg = 2.25), followed by **7a** (Δlg = 2.0), and slightly weaker for **6b** (Δlg = 1.75) (Figure 3c, Table 3). The activity was significantly weaker for isomers **5a** and **5b**. The effect was again time-dependent, but in all three viral strains studied, even at the last time interval (120 min), the inhibition was weak (Table 3).

### 2.4. Molecular Docking Studies

Molecular docking studies were conducted to elucidate the potential conformations of the synthesized camphor-based molecules, which possessed virucidal and adsorption-inhibitory activities against multiple viruses. The docking studies focused on key viral glycoproteins, capsid proteins, and host receptors (HSV-1 gC from the viral envelope, HCoV-OC43 spike protein, FCV capsid P domain) to gain molecular-level insights into the interactions responsible for the observed antiviral activities. Site-directed docking was conducted at literature-validated functional sites lacking co-crystallized ligands, which precluded redocking-based validation. Docking grids were centered on key residues, while blind docking was not conducted due to computational constraints.

#### 2.4.1. Docking in HSV-1 gC from the Viral Envelope (PDB: **9Q9L**)

The choice of the HSV-1 prefusion glycoprotein B (PDB: **9Q9L**) as the docking target in this study is based on its crucial role as a class III viral fusion protein responsible for mediating the fusion of the viral envelope with the host cell membrane during viral entry. The docking grid was specifically centered on residue 255—located within the fusion loop region—which is a key functional epitope involved in initiating membrane fusion. Residue 255 (Chain A) also lies within the nanobody-binding epitope that maintains the metastable prefusion conformation, as described by Vollmer et al. [33].

Four ligands (**6a**, **6b**, **7a**, and **7b**) were used, and their binding potential against HSV1 glycoprotein B was evaluated. The calculated binding affinities ranged from −2.55 to −3.75 kcal/mol, indicating moderate to weak binding (Table 4).

**Figure 4:**
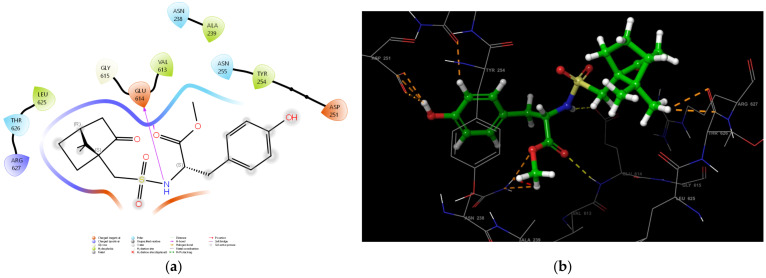

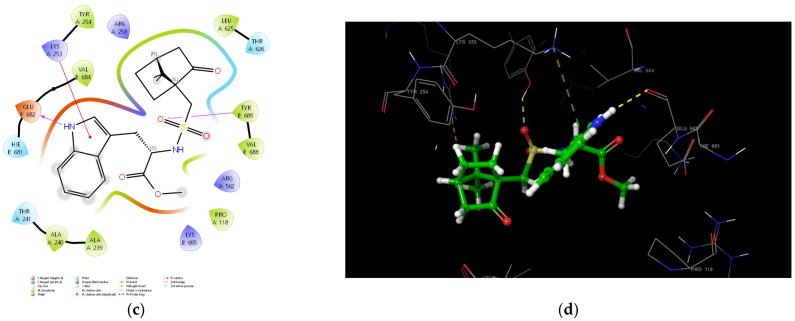
Molecular interactions of compound 6a (**a**,**b**) and the most active compound 7a (**c**,**d**) with HSV-1 glycoprotein B (PDB ID: **9Q9L**).

Despite the modest docking scores, the observed residue-level interactions provide meaningful insights into ligand stabilization and potential active conformations. Compound **6a** formed a single hydrogen bond with Glu614 (2.06 Å) (Figure 4a,b). Compound **6b** established two hydrogen bonds with Asp251 (2.23 Å) and Glu614 (2.07 Å). The involvement of acidic residues suggests stabilization through donor–acceptor hydrogen bonding, yet the moderate docking score suggests a binding orientation in a more solvent-exposed area of the protein. Ligand **7a** displayed the most favorable energy (−3.75 kcal/mol) and the richest interaction pattern: it formed a π-cation interaction with Lys253 (4.84 Å) and hydrogen bonds with Glu682 (2.05 Å) and Tyr689 (1.86 Å) (Figure 4c,d). However, it should be noted that the GlideXP scores were low to moderate. The combination of electrostatic and hydrogen-bonding forces indicates favorable interactions, possibly involving functionally significant residues within the fusion domain. **7b** showed a low binding energy of −2.55 kcal/mol and interacted with Pro236 (2.18 Å) and Glu614 (2.27 Å) through hydrogen bonds.

#### 2.4.2. Docking in HCoV-OC43 Spike Protein Structure (PDB: **9BLK**)

**Figure 5:**
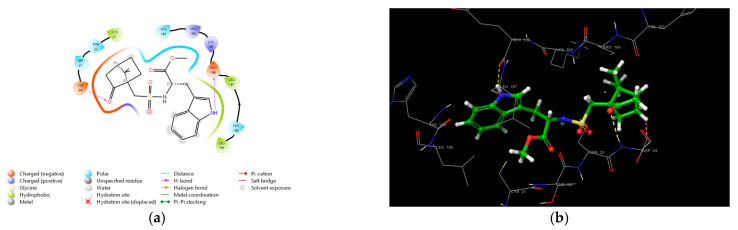

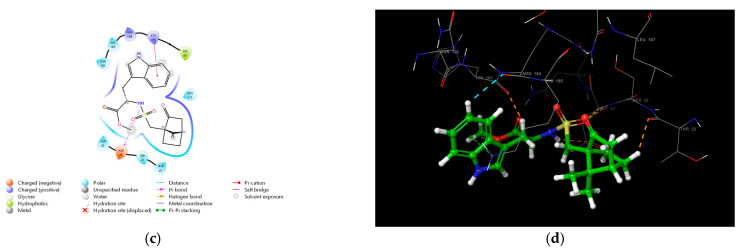
Molecular interactions of 7a (**a**,**b**) and 7b (**c**,**d**) in HCoV-OC43 spike protein (PDB: **9BLK**).

The HCoV-OC43 spike protein facilitates viral attachment to host cells through binding to 9-O-acetylated sialic acid receptors located in the N-terminal domain (NTD) of the spike S1 subunit. The crystal structure (PDB: **9BLK**) reveals a surface-exposed binding pocket where residues such as Tyr184 play a key role in recognizing these sialylated glycans [34]. We centered the docking grid on Tyr184 within this NTD region to target the sialic acid binding site. This approach aims to identify how the compounds might block viral adsorption by interfering with spike–receptor interactions, consistent with its biological effects on virus–cell attachment. The docking results show interactions between the **7a**, Figure 5a,b, and **7b**, Figure 5c,d, and key residues within the HCoV-OC43 spike protein’s NTD, focusing on the sialic acid binding site. Moderate binding affinities were noted, with a score of −3.08 kcal/mol for **7a** corresponding to interactions primarily with Asp24 and Glu186 (Table 4). These residues form hydrogen bonds with the ligand at distances of 2.64 Å and 2.04 Å, respectively. **7b** compound exhibits a Glide score of −2.58 kcal/mol, interacting with Asp24 through two hydrogen bonds (1.77 Å and 1.71 Å) and engaging Lys185 via a πι-cation interaction at 3.63 Å. These interactions collectively suggest a diverse binding mode that might interfere with the spike protein’s recognition of the 9-O-acetylated sialic acid receptors.

#### 2.4.3. Docking in FCV Capsid Protein P Domain (PDB: **6GSH**)

**Figure 6:**
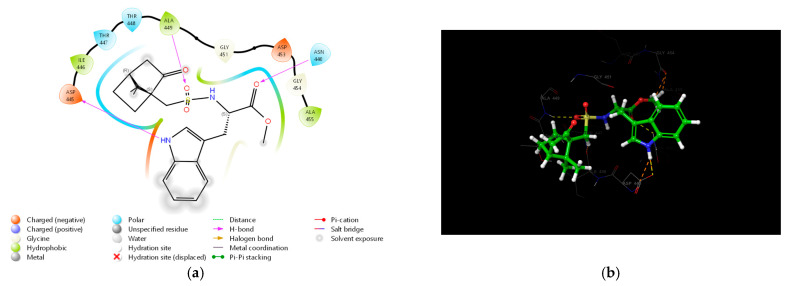

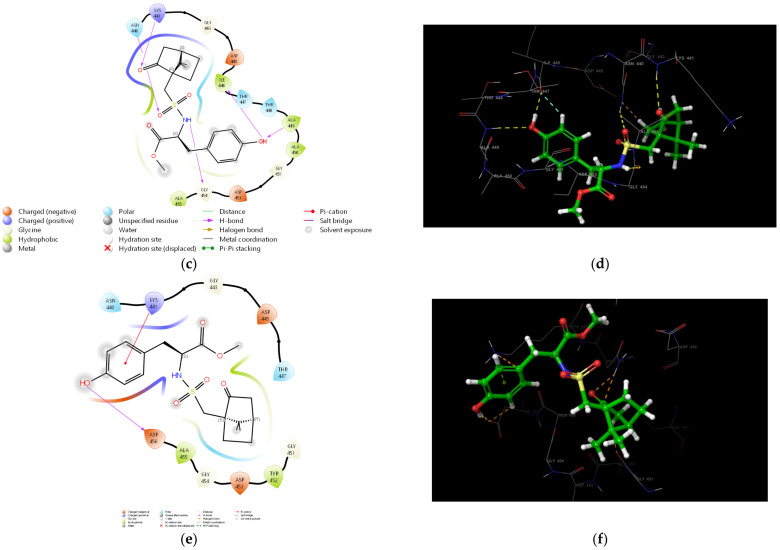
Molecular interactions of 7a (**a**,**b**), 6b (**c**,**d**), and 6a (**e**,**f**) in FCV capsid protein P domain (PDB: **6GSH**).

Experimental data have shown that some compounds exhibit virucidal activity and inhibit FCV adsorption in CRFK cells, where viral attachment is mediated by interactions between the viral capsid protein VP1 and host cell receptors. Since PDB: **6GSH** captures the viral capsid protein–receptor complex, it provides a relevant structural framework for analyzing and predicting the binding of compounds that may disrupt this critical virus–cell interaction, thereby blocking viral entry and adsorption [35]. The docking scores suggested moderate binding affinities for all three compounds, with **7a** exhibiting the most favorable score of −4.27 kcal/mol, followed by **7a** (−3.36 kcal/mol) and **6a** (−3.08 kcal/mol) (Table 4). However, the docking scores are still classified as low to moderate. Analysis of the binding interactions revealed that **6b** formed multiple hydrogen bonds with critical residues, including Asn440 (2.15 Å), Lys441 (2.74 Å), Ile446 (1.86 Å), Ala449 (2.71 Å), and Gly454 (2.06 Å), Figure 6c,d. These interactions might contribute to the slightly enhanced binding affinity observed for **6b**. Hydrogen bonding with Lys441 and Asn440, located within the P domain, suggests potential stabilization roles in ligand binding. **7a** exhibited several hydrogen bonds with Asp445 (1.59 Å) and Ala449 (2.28 Å), Figure 6a,b, indicating specific polar interactions within the binding site that likely underpin its moderate binding affinity. Although fewer interactions were observed relative to **6b**, these hydrogen bonds are significant for the ligand stabilization. **6a**, with the weakest docking score, demonstrated a π-cation interaction with Lys441 (3.23 Å) and a hydrogen bond with Asp456 (1.68 Å), Figure 6e,f. Several hydrogen bonds involving residues Asn440, Asp445, Ala449, and Gly454 were also observed.

The relatively weak binding scores of **7a** in the selected targets prompted more robust IFD simulations with MM/GBSA rescoring (Table 5). The results showed some improvements in predicted binding affinities. The IFD, followed by MM/GBSA rescoring of compound **7a** against the three viral targets, confirmed minor differences in the active conformations from the initial Glide SP docking predictions.

For the HCoV-OC43 spike protein (PDB: **9BLK**), the Glide score of −3.08 kcal/mol refined to an IFD score of −4.14 kcal/mol and MM/GBSA binding free energy of −38.68 kcal/mol. Similarly, for FCV capsid P domain (PDB: **6GSH**), Glide’s −3.36 kcal/mol improved to IFD −3.41 kcal/mol and MM/GBSA −35.50 kcal/mol, while in HSV-1 glycoprotein B (PDB: **9Q9L**), the progression from Glide −3.75 kcal/mol to IFD −3.47 kcal/mol and MM/GBSA −41.49 kcal/mol further validated the binding mode.

These results demonstrate pose reproducibility across methods, as IFD maintains Glide geometries while introducing receptor–side chain flexibility to resolve minor steric clashes, yielding slightly refined docking scores. The dramatic MM/GBSA improvements (from ~−3 to −4 kcal/mol to −35 to −41 kcal/mol) arise from explicit solvation, van der Waals contributions, and ligand desolvation penalties, providing a more physiologically relevant ΔG_bind estimate that underscores **7a**’s high-affinity binding potential at functional sites that are critical for viral adsorption and virucidal activity.

Depending on the introduction of different substituents into the sulfonamide structure, the antiviral activity of the parent molecule can be enhanced or impaired. The synthesis of a series of (*S*)-(+)-camphor-10-sulfonamides containing different substituents at the nitrogen atom has been described. Using a pseudovirus system based on the vesicular stomatitis virus (VSV), their antiviral activity against the Ebola virus is determined. Their antiviral activity increases significantly when there is a carbonyl group at the C-2 atom. On the other hand, transformation into an oxime leads to the almost complete disappearance of antiviral activity. The interactions of the studied sulfonamides with the Ebola virus glycoprotein have also been studied by molecular modeling [24].

Based on the presented results, the camphor-based sulfonamides studied in this work most likely interact with viral surface structures involved in the attachment of the virion to the host cell. This assumption is supported by the results of our antiviral experiments and molecular docking analyses, which consistently indicate interference with adsorption-related viral components as the main mechanism of action. This effect is mostly pronounced for compounds **7a** and **7b**, which showed activity against all three viral models studied, indicating that the presence of the tryptophan residue in the molecule significantly enhances antiviral potency. Molecular docking shows that compound **7a** binds to key viral proteins and host receptors, forming important hydrogen bonds and electrostatic interactions with residues like Lys253 and Glu614 in HSV-1 glycoprotein B, as well as critical sites on the HCoV-OC43 spike protein. Compounds **6a** and **6b** show a weaker effect, most notably against HSV-1 and to a lesser degree against FCV, suggesting that the inclusion of a tyrosine residue also contributes to improved antiviral properties. In contrast, the glycine-based derivatives **5a** and **5b** show no appreciable antiviral activity. These findings, consistent with previous observations on related camphor-based sulfonamides [36], suggest that the antiviral activity might be associated with surface-level interactions that interfere with viral adsorption.

## 3. Materials and Methods

### 3.1. General Information

All reagents were purchased from Aldrich (Wyoming, IL, USA), Merck (Darmstadt, Germany), and Fluka (Buchs, Switzerland) and were used without further purification. Thin-layer chromatography (TLC) was carried out on aluminum plates precoated with silica gel 60 F_254_ (Merck, Darmstadt, Germany), flash column chromatography was performed using silica gel 60 (230–400 mesh, Merck, Rahway, NJ, USA). The deuterated solvents were supplied by Deutero GmbH (Kastellaun, Germany). Melting points were determined in capillary tubes using a UniMelt 3 automated system (LLG Labware, Meckenheim, Germany). Optical rotation values [α]_D_^20^ were measured with a JASCO P-2000 polarimeter (Jasco Inc., Hachioji, Japan). The ^1^H and ^13^C NMR spectra were recorded on a Bruker Avance NEO 400 spectrometer (Bruker, Rheinstetten, Germany) in CDCl_3_ or DMSO-d_6_ with TMS as the internal reference (δ, ppm). The data included chemical shifts, multiplicities (s = singlet, d = doublet, t = triplet, q = quartet, br = broad, m = multiplet), integration values, coupling constants (*J*, Hz), and signal assignments. The assignments of the ^1^H and ^13^C NMR spectra were established based on DEPT, COSY, HSQC, and HMBC experiments. The NMR spectra were processed with the Topspin 4.1.4 software program (Bruker, Rheinstetten, Germany). High-resolution mass spectra (HRMS) were recorded on a Q Exactive Plus Hybrid Quadrupole-Orbitrap Mass Spectrometer (Thermo Fisher Scientific Inc., Waltham, MA, USA, HESI HRMS) in positive and/or negative mode. Spectra were processed by the Thermo Scientific FreeStyle program version 1.8 SP1 (Thermo Fisher Scientific Inc., Waltham, MA, USA).

### 3.2. General Procedure and Characterization Data for Camphor-10-Sulfonamides (***5a,b***–***7a,b***)

Method A: To a stirred solution of the corresponding amino acid methyl ester hydrochloride (**2**–**4**, 2.36 mmol) in dry CH_2_Cl_2_ or MeOH (6 mL) at 0 °C, Et_3_N (2.36 mmol) was added dropwise, followed by the portionwise addition of (*S*)- or (*R*)-camphorsulfonyl chloride (**1** or **2**, 1.97 mmol). The reaction mixture was stirred at 0 °C for 30 min, then allowed to reach room temperature and stirred for 24 h. The reaction progress was monitored by TLC (hexane/ethyl acetate 4:1). The mixture was washed with water. The organic layer was dried over anhydrous Na_2_SO_4_ and concentrated under reduced pressure. The crude residue was purified by flash chromatography (silica gel, hexane/ethyl acetate 4:1, 2:1, 1:1) to afford the target camphor-10-sulfonamide derivatives (**5a**,**b**–**7a**,**b**) as white crystalline solids.

Method B: The reactions were also carried out in aqueous medium using Na_2_CO_3_ (1.99 mmol) as a base. To a stirred aqueous solution (6 mL) of the corresponding amino acid methyl ester hydrochloride (**2**–**4**, 1.99 mmol) at 0 °C, the (*S*)- or (*R*)-camphorsulfonyl chloride (**1** or **2**, 1.99 mmol) was added in small portions. The reaction mixture was stirred at 0 °C for 30 min and then at room temperature for 24 h. After completion, the mixture was acidified to pH 2.5 with 2N HCl, the solvent was evaporated under reduced pressure, and the residue was purified by flash chromatography (hexane/ethyl acetate 4:1, 2:1, 1:1) to afford the target camphor-10-sulfonamide derivatives (**5a**,**b**–**7a**,**b**) as white crystalline solids.

Compound **5a** (methyl (*S*)-2-((((1*S*,4*R*)-7,7-dimethyl-2-oxobicyclo[2.2.1]heptan-1-yl)methyl)sulfonamido)-2-phenylacetate): colorless solid; yield 73% (method A), 13% (method B); m.p. 90.5–92 °C; [α]_D_^20^ = +84.0 (*c* = 1.0, CHCl_3_); ^1^H NMR (400 MHz, CDCl_3_): δ ppm 0.93 (s, 3H, H-8), 0.99 (s, 3H, H-9), 1.41–1.47 (m, 1H, H-5), 1.93 (d, *J* = 18.8 Hz, 1H, H-3), 1.91–2.11 (m, 4H, H-4, H-5, H-6), 2.39–2.46 (m, 1H, H-3), 2.94 (d, *J* = 15.0, 1H, H-10), 3.56 (d, *J* = 15.0, 1H, H-10), 3.73 (s, 3H, H-13), 5.31 (d, *J* = 7.9 Hz, 1H, H-11), 6.58 (d, *J* = 7.9 Hz, 1H, NH), 7.32–7.42 (m, 5H, H-15, H-16, H-17, H-19, H-19); ^13^C NMR (CDCl_3_): δ ppm 19.50 (q, C-9), 19.91 (q, C-8), 26.67 (t, C-6), 27.06 (t, C-5), 42.80 (d, C-4), 42.93 (t, C-3), 48.78 (s, C-7), 52.00 (t, C-10), 59.27 (q, C-13), 60.12 (d, C-11), 60.40 (s, C-1), 127.42 (2d, C-15, C-19), 128.77 (1C, C-17), 129.06 (2d, C-16, C-18), 136.08 (s, C-14), 171.40 (s, C-12), 216.40 (s, C-2); HRMS (HESI) m/z calcd. for C_19_H_25_NO5SH^+^ [M+H]^+^ = 380.1527, found 380.1523, Δ = −1.0 ppm; m/z calcd. for C_19_H_25_NO5SH^−^ [M+H]^−^ = 378.1381, found 378.1385, Δ = 1.1 ppm.

Compound **5b** (methyl (*S*)-2-((((1*R*,4*S*)-7,7-dimethyl-2-oxobicyclo[2.2.1]heptan-1-yl)methyl)sulfonamido)-2-phenylacetate): colorless solid; yield 70% (method A), 21% (method B); m.p. 93.5–95 °C; [α]_D_^20^ = +81.0 (*c* = 1.0, CHCl_3_); ^1^H NMR (400 MHz, CDCl_3_): δ ppm 0.47 (s, 3H, H-8), 0.78 (s, 3H, H-9), 1.30–1.36 (m, 1H, H-5), 1.82 (d, *J* = 18.6 Hz, 1H, H-3), 2.05–1.85 (m, 4H, H-4, H-5, H-6), 2.22–2.29 (m, 1H, H-3), 2.87 (d, *J* = 13.5, 1H, H-10), 2.95 (d, *J* = 13.5, 1H, H-10), 3.68 (s, 3H, H-13), 5.20 (d, *J* = 8.2 Hz, 1H, H-11), 6.55 (d, *J* = 8.4 Hz, 1H, NH), 7.36–7.25 (m, 5H, H-15, H-16, H-17, H-19, H-19); ^13^C NMR (CDCl_3_): δ ppm 19.35 (q, C-9), 19.70 (q, C-8), 26.97 (t, C-6), 27.10 (t, C-5), 42.69 (d, C-4), 42.85 (t, C-3), 48.52 (s, C-7), 52.48 (t, C-10), 53.03 (q, C-13), 59. (s, C-1), 60.17 (d, C-11), 127.95 (2d, C-15, C-19), 128.99 (1C, C-17), 129.18 (2d, C-16, C-18), 135.85 (s, C-14), 170.34 (s, C-12), 216.25 (s, C-2); HRMS (HESI) m/z calcd. for C_19_H_25_NO_5_SH^+^ [M+H]^+^ = 380.1526, found 380.1525, Δ = −0.3 ppm; m/z calcd. for C_19_H_25_NO5SH^−^ [M+H]^−^ = 378.1381, found 378.1385, Δ = 1.1 ppm.

Compound **6a** (methyl ((((1*S*,4*R*)-7,7-dimethyl-2-oxobicyclo[2.2.1]heptan-1-yl)methyl)sulfonyl)-L-tyrosinate): colorless solid; yield 17% (method A); 15% (method B); m.p. 117.8–119.9 °C; [α]_D_^20^ = +15 (*c* = 1.03, CHCl_3_); ^1^H NMR (400 MHz, CDCl_3_): δ ppm 0.86 (s, 3H, H-8), 0.97 (s, 3H, H-9), 1.38–1.44 (m, 1H, H-5), 1.87–1.94 (m, 2H, H-3, H-6), 1.95–2.04 (m, 1H, H-5), 2.08–2.19 (m, 2H, H-4, H-6), 2.33–2.40 (m, 1H, H-3), 2.88 (d, *J* = 15.0 Hz, 1H, H-10), 3.00–3.12 (m, 2H, H-14), 3.40 (d, *J* = 15.0 Hz, 1H, H-10), 3.73 (s, 3H, H-13), 4.46–4.51 (m, 1H, H-11), 6.01 (d, *J* = 9.1 Hz, 1H, NH), 6.71–6.74 (m, 2H, H-17, H-19), 7.03–7.06 (m, 2H, H-16, H-20); ^13^C NMR (400 MHz, CDCl_3_): δ ppm 19.48 (q, C-9), 19.86 (q, C-8), 26.55 (t, C-6), 27.00 (t, C-5), 38.77 (t, C-14), 42.75 (d, C-4), 42.85 (t, C-3), 48.67 (s, C-7), 51.57 (t, C-10), 52.48 (q, C-13), 57.60 (d, C-11), 59.13 (s, C-1), 115.62 (2d, C-17, C-19), 127.12 (s, C-15), 130.64 (2d, C-16, C-20), 155.19 (s, C-18), 172.21 (s, C-12), 216.48 (s, C-2); HRMS (HESI) m/z calcd. for C_20_H_27_NO_6_SH^+^ [M+H]^+^ = 410.1632, found 410.1637, Δ = 0.0 ppm; m/z calcd. for C_20_H_27_NO_6_SH^−^ [M+H]^−^ = 408.1486, found 408.1493, Δ = 1.7 ppm.

Compound **6b** (methyl ((((1*R*,4*S*)-7,7-dimethyl-2-oxobicyclo[2.2.1]heptan-1-yl)methyl)sulfonyl)-L-tyrosinate): colorless solid; yield 38% (method A); 10% (method B); m.p. 116.5–117.2 °C; [α]_D_^20^ = −20.0 (*c* = 1.2, CHCl_3_); ^1^H NMR (400 MHz, CDCl_3_), δ ppm 0.79 (s, 3H, H-8), 0.99 (s, 3H, H-9), 1.36–1.43 (m, 1H, H-5), 1.71–1.78 (m, 1H, H-6), 1.91 (d, *J* = 18.5 Hz, 1H, H-3), 1.95–2.04 (m, 1H, H-5), 2.07 (t, *J* = 4.5 Hz, 1H, H-4), 2.25–2.39 (m, 2H, H-3, H-6), 2.67 (d, *J* = 14.9 Hz, 1H, H-10), 2.94–2.99 (m, 1H, H-14), 3.07–3.12 (m, 1H, H-14), 3.25 (d, *J* = 14.9 Hz, 1H, H-10), 3.76 (s, 3H, H-13), 4.41–4.46 (m, 1H, H-11), 5.42 (d, *J* = 8.8 Hz, 1H, NH), 5.88 (br s, 1H, OH), 6.73–6.76 (m, 2H, H-17, H-19), 7.02–7.06 (m, 2H, H-16, H-20); ^13^C NMR (400 MHz, CDCl_3_): δ ppm 19.62 (q, C-9), 19.78 (q, C-8), 25.65 (t, C-6), 26.89 (t, C-5), 38.60 (t, C-14), 42.72 (t, C-3), 42.74 (d, C-4), 48.27 (s, C-7), 51.12 (t, C-10), 52.71 (q, C-13), 57.38 (d, C-11), 58.74 (s, C-1), 115.69 (2d, C-17, C-19), 127.25 (s, C-15), 130.71 (2d, C-16, C-20), 155.22 (s, C-18), 172.25 (s, C-12), 215.89 (s, C-2); HRMS (HESI) m/z calcd. for C_20_H_27_NO_6_SH^+^ [M+H]^+^ = 410.1632, found 410.1631, Δ = −0.2 ppm; m/z calcd. for C_20_H_27_NO_6_SH^−^ [M+H]^−^ = 408.1486, found 408.1491, Δ = 1.2 ppm.

Compound **7a** (methyl ((((1*S*,4*R*)-7,7-dimethyl-2-oxobicyclo[2.2.1]heptan-1-yl)methyl)sulfonyl)-L-tryptophanate): colorless solid; yield 18% (method A); 17% (method B); m.p. 207.1–208.5; [α]_D_^20^ = +31.0 (*c* = 1.0, CHCl_3_); ^1^H NMR (400 MHz, CDCl_3_): δ ppm 0.76 (s, 3H, H-8), 0.89 (s, 3H, H-9), 1.28–1.34 (m, 1H, H-5), 1.77–1.84 (m, 2H, H-3, H-6), 1.86–1.95 (m, 1H, H-5), 1.99 (t, *J* = 8.8 Hz, 1H, H-4), 2.04–2.11 (m, 1H, H-6), 2.24–2.31 (m, 1H, H-3), 2.80 (d, *J* = 15.0 Hz, 1H, H-10), 3.22–3.32 (m, 2H, H-14), 3.36 (d, *J* = 15.0 Hz, 1H, H-10), 3.59 (s, 3H, H-13), 4.49–4.55 (m, 1H, H-11), 5.98 (d, *J* = 8.9 Hz, 1H, NH), 7.02–7.11 (m, 3H, H-18, H-19, H-22), 7.26 (d, *J* = 8.0 Hz, 1H, H-20), 7.51 (d, *J* = 7.6 Hz, 1H, H-17), 8.10 (br s, 1H, NH); ^13^C NMR (400 MHz, CDCl_3_): δ ppm 19.50 (q, C-9), 19.86 (q, C-8), 26.58 (t, C-6), 27.01 (t, C-5), 29.47 (t, C-14), 42.76 (d, C-4), 42.85 (t, C-3), 48.62 (s, C-7), 51.62 (t, C-10), 52.50 (q, C-13), 56.88 (d, C-11), 59.17 (s, C-1), 109.58 (s, C-15), 111.26 (d, C-20), 118.65 (d, C-17), 119.63 (d, C-18), 122.15 (d, C-19), 123.36 (d, C-22), 127.42 (s, C-16), 136.10 (s, C-21), 172.35 (s, C-12), 216.05 (s, C-2); HRMS (HESI) m/z calcd. for C_22_H_28_N_2_O_5_SH^+^ [M+H]^+^ = 433.1792, found 433.1791, Δ = −0.2 ppm; m/z calcd. for C_22_H_28_N_2_O_5_SH^−^ [M+H]^−^ = 431.1646, found 431.1646, Δ = 0 ppm.

Compound **7b** (methyl ((((1*R*,4*S*)-7,7-dimethyl-2-oxobicyclo[2.2.1]heptan-1-yl)methyl)sulfonyl)-L-tryptophanate): colorless solid; yield 26% (method A); 16% (method B); m.p. 207.8–209.0 °C; [α]_D_^20^ = −25.0 (*c* = 1.3, CHCl_3_); ^1^H NMR (400 MHz, CDCl3) δ, ppm: 0.59 (s, 3H, H-8), 0.90 (s, 3H, H-9), 1.32–1.38 (m, 1H, H-5), 1.69–1.76 (m, 1H, H-6), 1.85 (d, *J* = 18.4 Hz, 1H, H-3), 1.90–1.99 (m, 1H, H-5), 2.02 (t, *J* = 4.5 Hz, 1H, H-4), 2.21–2.31 (m, 2H, H-3, H-6), 2.58 (d, *J* = 14.9 Hz, 1H, H-10), 3.17 (d, *J* = 14.9 Hz, 1H, H-10), 3.22–3.28 (m, 1H, H-14), 3.34–3.39 (m, 1H, H-14), 3.74 (s, 3H, H-13), 4.51–4.56 (m, 1H, H-11), 5.49 (d, *J* = 8.1 Hz, 1H, NH), 7.09–7.19 (m, 3H, H-18, H-19, H-22), 7.32–7.35 (m, 1H, H-20), 7.58–7.60 (m, 1H, H-17), 8.22 (s, 1H, NH); ^13^C NMR (400 MHz, CDCl_3_): δ ppm 19.50 (q, C-9), 19.52 (q, C-8), 25.64 (t, C-6), 26.89 (t, C-5), 29.26 (t, C-14), 42.67 (t, C-3), 42.69 (d, C-4), 48.06 (s, C-7), 50.74 (t, C-10), 52.68 (q, C-13), 56.44 (d, C-11), 58.63 (s, C-1), 109.42 (s, C-15), 111.41 (d, C-20), 118.57 (d, C-17), 119.81 (d, C-18), 122.34 (d, C-19), 123.80 (d, C-22), 127.14 (s, C-16), 136.23 (s, C-21), 172.46 (s, C-12), 215.42 (s, C-2); HRMS (HESI) m/z calcd. for C_22_H_28_N_2_O_5_SH^+^ [M+H]^+^ = 433.1792, found 433.1788, Δ = 0.9 ppm; m/z calcd. for C_22_H_28_N_2_O_5_SH^−^ [M+H]^−^ = 431.1646, found 431.1646, Δ = 0 ppm.

### 3.3. Host Cell Lines

Madin–Darbey bovine kidney (MDBK) cells, Crandell–Rees Feline Kidney (CRFK) cells, and Vero E6 (isolated from the kidney of an African green monkey) cells were provided by the National Bank for Industrial Microorganisms and Cell Cultures, Sofia, Bulgaria. Cells were grown in DMEM growth medium (Gibco, Grand Island, NY, USA) containing 10% fetal bovine serum (Gibco, Grand Island, NY, USA), supplemented with 10 mM HEPES buffer (AppliChem GmbH, Darmstadt, Germany) and antibiotics (penicillin 100 IU/mL, streptomycin 100 μg/mL). Incubation was performed in an incubator (HERA cell 150, Heraeus, Hanau, Germany) at 37 °C and a 5% CO_2_ atmosphere.

### 3.4. Viruses

Human Coronavirus OC-43 (HCoV-OC43) (ATCC, Manassas, VA, USA; VR-1558) strain was propagated in Vero E6 cells in DMEM supplemented with 2% fetal bovine serum, 100 U/mL penicillin, and 100 μg/mL streptomycin. Cells were lysed 5 days after infection by 2 freeze and thaw cycles, and the virus was titrated according to the Reed and Muench formula [37]. Virus and mock aliquots were stored at −80 °C. The infectious titer of the stock virus was 10^6.5^CCID_50_/mL.

Herpes simplex virus type 1, Victoria strain (HSV-1) was received from Prof. S. Dundarov, National Center of Infectious and Parasitic Diseases, Sofia. The virus was replicated in a confluent monolayer of MDBK cells.

Feline calicivirus (FCV) (from the collection of the Institute of Microbiology, Bulgarian Academy of Sciences) was propagated in cells of the CRFK cell line. In the case of HSV and FCV, the infected cells were incubated in the presence of Dulbecco’s modified Eagle’s medium (DMEM), Gibco BRL, Paisley, Scotland, UK, plus 0.5% fetal bovine serum (Gibco BRL, Scotland, UK) and antibiotics (penicillin 100 IU/mL, streptomycin 100 μg/mL). After incubation at 37 °C in a 5% CO_2_ incubator, the viral yield was frozen at −80 °C. The infectious viral titer of the initial virus stock was 10^8.5^ CCID_50_/mL for HSV and 10^7.25^ CCID_50_/mL for FCV.

### 3.5. Reference Compound

Remdesivir (GS-5734, RDV, REM, Veklury^®^) (Gilead Science Ireland UC) was initially dissolved in double-distilled water to a concentration of 150 mg/mL and then diluted in DMEM nutrient medium to the required concentrations.

Acyclovir {ACV, [9-(2-hydroxyethoxymethyl)-guanine]} was kindly provided by the Deutsches Kresforschung Zentrum, Heidelberg, with a stock concentration of 3 mM solution in DMSO. Then, serial dilutions were made in DMEM to the required concentrations.

### 3.6. Cytotoxicity Assay

A confluent monolayer cell culture in 96-well plates (Costar^®^, Corning Inc., Kennebunk, ME, USA) was treated with 0.1 mL/well containing support medium that did not contain or contained decreasing concentrations of the tested substances. Cells were incubated under the characteristic conditions under which subsequent virus experiments would be performed. MDBK and CRFK cells were incubated with the substances for 48 h, and Vero E6 cells for 120 h at 37 °C and 5% CO_2_. After the given period of time, the tested extracts were removed, and the cells were washed and incubated with neutral red (NR) dye at 37 °C for 3 h. The 50% cytotoxic concentration (CC_50_) was defined as the concentration of the extract that reduced cell viability by 50% compared to untreated controls [38]. Each compound was tested in triplicate, with four wells per replicate. The maximally tolerated concentration (MTC) of the tested samples, at which they do not affect the morphology of the cell monolayer, was also determined. Each experiment was repeated 4 times (*n* = 4).

### 3.7. Determination of Infectious Viral Titers

In 96-well plates, a monolayer of Vero E6, MDBK, or CRFK cells was infected with 0.1 mL of virus suspension at tenfold decreasing dilutions [39]. After the virus adsorption period, the unabsorbed virus was removed, and the DMEM was added. This was followed by incubation at 37 °C and 5% CO_2_ in a HERA cell 150 CO_2_ incubator (Radobio Scientific Co., Ltd., Shanghai, China) for 48 h (for HSV-1 and FCV) and 120 h (for HCoV-OC43). Cells infected with the maximum concentration of the virus and demonstrating the maximum cytopathic effect were used as controls. The resulting cytopathic effect (CPE) was monitored by microscopic observation of the cell monolayer and confirmed by neutral red uptake assay [38]. Each experiment was repeated 4 times (*n* = 4).

### 3.8. Antiviral Activity Assay

The cytopathic effect inhibition (CPE) test was used to determine the antiviral activity of the tested substances [38]. A confluent cell monolayer in 96-well plates was infected with 100 cell culture infectious doses of 50% (CCID_50_) in 0.1 mL. After 2 h of adsorption (for HCoV-OC43) and 1 h of adsorption at 37 °C (for HSV-1 and FCV), unattached virus was removed and the tested sample was added at different concentrations and the cells were incubated for 5 days (for HCoV-OC43) or 2 days at 37 °C (for HSV-1 and FCV) and in the presence of 5% CO_2_. The cytopathic effect was determined using a neutral red uptake assay, and the percentage of CPE inhibition for each test sample concentration was calculated using the following formula:

%CPE = [OD_test sample_ − OD_virus control_]/[OD_toxicity control_ − OD_virus control_] × 100

where OD_test sample_ is the mean of the ODs of the wells inoculated with virus and treated with the tested substances at the corresponding concentration, ODs_virus control_ is the mean of the ODs of the virus control wells (no compound in the medium), and OD control for toxicity is the mean of the ODs of the wells not inoculated with virus but treated with the corresponding concentration of the substances. Each experiment was repeated four times (*n* = 4).

### 3.9. Effect on Viral Adsorption

Twenty-four-well plates containing a monolayer of VeroE6, MDBK, or CRFK cells were pre-chilled to 4 °C and inoculated with 10^4^ CCID_50_ of HCoV-OC43, HSV-1, or FCV, respectively. Along with the virus, the monolayer was also treated with the tested samples in their MTC and incubated at 4 °C for the time of virus adsorption. At different time intervals different for the types of virus (15, 30, 45, and 60 min for HSV-1 and FCV or 15, 30, 60, 90, and 120 min for HCoV-OC43), the virus and the tested sample were removed, the cells were washed with PBS, then the cells were covered with maintenance medium and incubated at 37 °C in the presence of 5% CO_2_ for 24 h. After freezing and thawing three times, the infectious viral titer of each sample was determined, compared to the viral control at the corresponding time interval, and the Δlg values were determined. Each sample was prepared in quadruplicate (*n* = 4).

### 3.10. Virucidal Assay

Samples with a total volume of 1 mL containing virus (10^5^ CCID_50_) and tested substances at their maximally tolerated concentration (MTC) in a 1:1 ratio were prepared. A sample containing the untreated virus diluted 1:1 with DMEM was incubated in parallel. Both the control and experimental samples were incubated at room temperature for different time intervals (15, 30, 60, 90, and 120 min). Then, according to the endpoint dilution method formula [37], the content of residual infectious virus in each sample was determined, compared with the viral control, and the Δlg values were calculated. Each experiment was repeated four times (*n* = 4).

### 3.11. Datasets Preparations

The newly synthesized camphor-based compounds were drawn using ChemDraw v25.0 and prepared for molecular docking with the LigPrep module in Schrödinger (Schrödinger Release 2025-1: LigPrep, Schrödinger, LLC, New York, NY, USA, 2025). The preparation steps included conversion to 3D structures, addition of missing hydrogen atoms, generation of ionization and tautomeric states at physiological pH (7.0 ± 0.2), assignment of bond orders, and energy minimization using the OPLS4 force field to optimize ligand geometries for docking studies.

### 3.12. Protein Preparation

The crystal structures of HCoV-OC43 spike protein (PDB: **9BLK**) [40], FCV capsid protein P domain (PDB: **6GSH**) [35], and HSV-1 glycoprotein B (PDB: **9Q9L**) [33] were retrieved from the Protein Data Bank (accesed on 14 October 2025 https://www.rcsb.org/) and prepared using the Protein Preparation Wizard in Maestro (Schrödinger Release 2025-1: Protein Preparation Wizard; Epik, Schrödinger, LLC, New York, NY, USA, 2025). Preparation included: addition of missing hydrogen atoms, assignment of protonation states at pH 7.0 ± 0.4 using PROPKA, deletion of non-essential water molecules beyond 5 Å from heteroatoms, filling of missing side chains using Prime, and energy minimization using the OPLS4 force field with convergence criteria of 0.3 Å heavy atom RMSD. Receptor grids were generated using Receptor Grid Generation with the following specifications: grid center positioned on key residues (Tyr184 for HCoV-OC43 spike, Asp445 for FCV, and Asn255 for HSV-1), inner box dimensions of 20 × 20 × 20 Å, outer box dimensions of 40 × 40 × 40 Å, and default van der Waals scaling factors.

### 3.13. Docking Simulations

The prepared camphor-based ligands were docked using Glide Extra precision (XP) mode with 10,000 poses generated per ligand during initial screening, up to 100 poses retained for energy minimization, a maximum of 10 poses output per ligand, and post-docking minimization with an OPLS4 force field [41]. For the top-ranked compound, **7a**, subsequent Induced Fit Docking (IFD) was performed using the Prime module in the Schrödinger Suite, allowing receptor–side chain flexibility within a 5 Å residue radius of the Glide XP pose, with 20 poses generated per ligand, van der Waals scaling at 0.5, and OPLS4 minimization until convergence. The top IFD poses were then rescored using Prime MM/GBSA, incorporating VSGB 2.0 solvation model, 1000 energy minimization steps with OPLS4 force fieldand explicit treatment of protein–ligand desolvation penalties to yield ΔG bind estimates. The simulations utilized the same receptor grids centered on key functional residues as described in the initial Glide docking workflow.

### 3.14. Statistical Analysis

Data on cytotoxicity and antiviral effects were analyzed statistically. The values of CC_50_ were presented as means ± SD. The significance of the differences between the cytotoxicity values of the tested samples and the reference substances, as well as the effect of the virucidal activity of the samples compared to that of 70% ethanol, was determined by Student’s *t*-test; *p*-values of < 0.05 were considered significant. The final data sets were analyzed with the Graph Pad Prism 4 software. Each experiment was repeated four times (*n* = 4).

## 4. Conclusions

A series of camphor-10-sulfonamide derivatives was synthesized through a feasible and sustainable approach using inexpensive, naturally derived precursors. Although yields were moderate, the method enabled access to structurally diverse, chiral sulfonamides suitable for biological screening. The camphor-based sulfonamides exhibited distinct virucidal activity and affected the viral adsorption stage. The most noticeable effects were observed for tryptophan derivatives (**7a** and **7b**), and slightly weaker effects were observed for tyrosine derivatives (**6a** and **6b**), while glycine derivatives (**5a** and **5b**) did not contribute to noticeable antiviral activity. Docking pose rankings showed a general agreement with the observed activity trends, suggesting possible directions for analog design and lead optimization. The diverse binding profiles indicate that these camphor-based derivatives may function through multiple mechanisms of action, including receptor blocking, viral protein destabilization, and capsid and envelope integrity disruption. Overall, this study demonstrates that the integration of practical synthesis, renewable materials, and biological evaluation provides an efficient route toward new camphor-based sulfonamide scaffolds with promising antiviral potential.

## Figures and Tables

**Table 1:** In vitro assessment of cytotoxicity of the samples.

Sample	Cytotoxicity (µg/mL) ± SD ^1^					
	MDBK		VeroE6		CRFK	
	CC_50_	MTC	CC_50_	MTC	CC_50_	MTC
**5a**	1178.7 ± 8.3 ***	200	726.5 ± 8.2 ***	200	1085.2 ± 10.3	200
**5b**	1015.4 ± 9.8 ***	200	681.8 ± 8.6 ***	200	997.7 ± 9.4	200
**6a**	1526.5 ± 11.2 ***	200	1026.0 ± 10.6 ***	200	1346.7 ± 12.3	200
**6b**	1523.2 ± 9.8 ***	200	890.4 ± 8.2 ***	200	1338 ± 10.7	200
**7a**	1900.8 ± 12.7 ***	200	1293.4 ± 11.7 ***	200	1815.7 ± 12.3	200
**7b**	1422.8 ± 11.8 ***	200	753.6 ± 8.4 ***	200	1345.2 ± 9.8	200
Acyclovir	291.0 ± 9.4	nd ^2^	nd	nd	nd	nd
Remdesivir	nd	nd	250.0 ± 4.3	nd	nd	nd

^1^ SD—Standard deviation; ^2^ nd—no data; *** *p* < 0.0001, when comparing the value of each sample with the corresponding reference substance for the given cell line, Student’s *t*-test. Each experiment was repeated 4 times (*n* = 4).

**Table 2:** Influence on the viral adsorption stage.

Viral Strain	Time	Δlg ± SD ^1^					
		5a	5b	6a	6b	7a	7b
HSV-1	15 min	0.5	1.0	0.5 ± 0.1	0.5 ± 0.2	1.0 ± 0.1	0.5 ± 0.1
	30 min	0.5	1.0	0.5 ± 0.1	0.5 ± 0.1	1.0 ± 0.2	0.5 ± 0.1
	45 min	0.5	1.25	0.5 ± 0.2	0.5 ± 0.1	1.0 ± 0.1	0.5 ± 0.1
	60 min	0.5	1.25	0.5 ± 0.1	0.5 ± 0.1	1.0 ± 0.2	0.5 ± 0.2
HCoV-OC43	15 min	1.0	1.0	0.5 ± 0.1	0.5 ± 0.1	1.0 ± 0.1	1.0 ± 0.1
	30 min	1.0	1.0	0.5 ± 0.1	0.5 ± 0.1	1.5 ± 0.1	1.5 ± 0.1
	60 min	1.0	1.25	0.5 ± 0.1	0.7 ± 0.1	1.75 ± 0.1	1.75 ± 0.1
	90 min	1.0	1.25	0.75 ± 0.1	0.75 ± 0.1	2.0 ± 0.2	2.0 ± 0.1
	120 min	1.0	1.25	0.75 ± 0.1	0.75 ± 0.1	2.0 ± 0.1	2.5 ± 0.1
FCV	15 min	1.25	1.5	0	0	1.0 ± 0.1	1.0 ± 0.1
	30 min	1.25	1.5	1.0 ± 0.1	1.0 ± 0.1	1.5 ± 0.1	1.0 ± 0.1
	45 min	1.25	1.5	1.5 ± 0.1	1.5 ± 0.1	1.75 ± 0.1	1.0 ± 0.1
	60 min	1.25	1.5	2.0 ± 0.1	1.75 ± 0.1	1.75 ± 0.1	1.0 ± 0.1

^1^ SD—Standard deviation. Each experiment was repeated 4 times (*n* = 4).

**Table 3:** Virucidal activity of the samples.

Viral Strain	Time	Δlg ± SD ^1^						
		5a	5b	6a	6b	7a	7b	70% Ethanol
HSV-1	15 min	0.75	1.0	2.75 ± 0.1 ***	2.0 ± 0.2 ***	2.75 ± 0.1 ***	2.75 ± 0.1 ***	5.0 ± 0.1
	30 min	0.75	1.0	2.75 ± 0.1 ***	2.5 ± 0.1 ***	2.75 ± 0.2 ***	2.75 ± 0.2 ***	5.0 ± 0.1
	60 min	1.0	1.0	3.0 ± 0.2 ***	3.0 ± 0.2 ***	2.75 ± 0.2 ***	3.0 ± 0.1 ***	5.0 ± 0.2
	90 min	1.0	1.0	3.0 ± 0.2 ***	3.0 ± 0.1 ***	2.75 ± 0.2 ***	3.0 ± 0.2 ***	5.0 ± 0.2
	120 min	1.0	1.0	3.0 ± 0.3 ***	3.0 ± 0.2 ***	2.75 ± 0.2 ***	3.0 ± 0.2 ***	5.0 ± 0.1
HCoV-OC43	15 min	0.5	1.0	0.5 ± 0.1 ***	0.5 ± 0.1 ***	1.0 ± 0.1 ***	1.0 ± 0.1 ***	5.0 ± 0.1
	30 min	1.0	1.0	0.5 ± 0.1 ***	0.5 ± 0.1 ***	1.5 ± 0.1 ***	1.5 ± 0.1 ***	5.0 ± 0.1
	60 min	1.0	1.0	0.5 ± 0.1 ***	0.7 ± 0.1 ***	1.75 ± 0.1 ***	1.75 ± 0.1 ***	5.0 ± 0.1
	90 min	1.25	1.0	0.75 ± 0.1 ***	0.75 ± 0.1 ***	2.0 ± 0.1 ***	2.0 ± 0.1 ***	5.0 ± 0.1
	120 min	1.25	1.0	0.75 ± 0.1 ***	0.75 ± 0.1 ***	2.0 ± 0.1 ***	2.5 ± 0.1 ***	5.0 ± 0.1
FCV	15 min	1.25	1.5	0	0	1.0 ± 0.1 ***	1.0 ± 0.1 ***	5.0 ± 0.1
	30 min	1.25	1.5	1.0 ± 0.1 ***	1.0 ± 0.1 ***	1.5 ± 0.1 ***	1.0 ± 0.1 ***	5.0 ± 0.1
	60 min	1.25	1.5	1.5 ± 0.1 ***	1.5 ± 0.1 ***	1.75 ± 0.1 ***	1.0 ± 0.1 ***	5.0 ± 0.1
	90 min	1.5	1.5	2.0 ± 0.1 ***	1.75 ± 0.1 ***	1.75 ± 0.1 ***	1.0 ± 0.1 ***	5.0 ± 0.1
	120 min	1.5	1.5	2.25 ± 0.1 ***	1.75± 0.1 ***	2.0 ± 0.1 ***	1.0 ± 0.1 ***	5.0 ± 0.1

^1^ SD—Standard deviation; *** *p* < 0.001, when comparing the value of each tested substance with the value of 70% ethanol for the corresponding time interval, Student’s *t*-test. Each experiment was repeated 4 times (*n* = 4).

**Table 4:** Docking scores and key amino acids involved in stabilization of the most prominent newly synthesized camphor-based ligands in HCoV-OC43 spike protein (PDB: **9BLK**), FCV capsid protein P domain (PDB: **6GSH**), and HSV-1 capsid (PDB: **9Q9L**).

Cmpd. ^1^	HCoV-OC43 Spike Protein (PDB: 9BLK) kcal/mol	Key Amino Acids Involved in Stabilization	FCV Capsid Protein P Domain (PDB: 6GSH) kcal/mol	Key Amino Acids Involved in Stabilization	HSV-1 (PDB: 9Q9L) kcal/mol	Key Amino Acids Involved in Stabilization
**6a**	nd ^2^	nd	−3.08	Lys441 (pi-cation (3.23 Å)); Asp456 (H-bond (1.68 Å))	−3.22	Glu614 (H-bond (2.06 Å))
**6b**	nd	nd	−4.27	Asn440 (H-bond (2.15 Å)); Lys441 (H-bond (2.74 Å)); Ile446 (H-bond (1.86 Å)); Ala449 (H-bond (2.71 Å)); Gly454 (H-bond (2.06 Å))	−2.59	Asp251 (H-bond (2.23 Å)); Glu614 (H-bond (2.07 Å))
**7a**	−3.08	Asp24 (H-bond (2.64 Å)), Glu186 (H-bond (2.04 Å))	−3.36	Asp445 (H-bond (1.59 Å)); Ala449 (H-bond (2.28 Å)).	−3.75	Lys 253 (Pi-cation (4.84 Å)); Glu682 (H-bond (2.05 Å)); Tyr689 (H-bond (1.86 Å));
**7b**	−2.58	Asp24 (H-bond (1.77 Å), H-bond (1.71 Å)); Lys185 (p-cation (3.63 Å))	nd	nd	−2.55	Pro236 (H-bond (2.18 Å)); Glu614 (2.27 Å))

^1^ Cmpd—Compound. ^2^ nd—not determined.

**Table 5:** IFD and MM/GBSA scores of **7a** in HCoV-OC43 spike protein, FCV capsid protein P domain, and HSV-1.

Compound	HCoV-OC43 Spike Protein (PDB: 9BLK) kcal/mol		FCV Capsid Protein P Domain (PDB: 6GSH) kcal/mol		HSV-1 (PDB: 9Q9L) kcal/mol	
	IFD	MM/GBSA	IFD	MM/GBSA	IFD	MM/GBSA
**7a**	−4.14	−38.68	−3.41	−35.50	−4.47	−41.49

## Data Availability

The original contributions presented in this study are included in the article/Supplementary Material. Further inquiries can be directed to the corresponding authors.

## Supplementary Materials

The following supporting information can be downloaded at: https://www.mdpi.com/article/10.3390/ijms27020616/s1. Original NMR spectra (Figures S1–S12); Original HESI HR-MS spectra (Figures S13–S23); All active conformations on the tested compounds in the target proteins (Figure S24).

## References

[1] Lozano R., Naghavi M., Foreman K., Lim S., Shibuya K., Aboyans V., Abraham J., Adair T., Aggarwal R., Ahn S.Y. (2012). Global and regional mortality from 235 causes of death for 20 age groups in 1990 and 2010: A systematic analysis for the Global Burden of Disease Study 2010. Lancet.

[2] De Clercq E. (2012). Milestones in the discovery of antiviral agents: Nucleosides and nucleotides. Acta Pharm. Sin. B.

[3] Sodano F., Gazzano E., Fruttero R., Lazzarato L. (2022). NO in Viral Infections: Role and Development of Antiviral Therapies. Molecules.

[4] García-Serradilla M., Risco C., Pacheco B. (2019). Drug repurposing for new, efficient, broad spectrum antivirals. Virus Res..

[5] Capasso C., Supuran C.T. (2015). An overview of the alpha-, beta-and gamma-carbonic anhydrases from Bacteria: Can bacterial carbonic anhydrases shed new light on evolution of bacteria?. J. Enzym. Inhib. Med. Chem..

[6] Supuran C.T. (2017). Carbonic Anhydrase Inhibition and the Management of Hypoxic Tumors. Metabolites.

[7] Masaret G.S. (2020). Synthesis, Docking and Antihypertensive Activity of Pyridone Derivatives. ChemistrySelect.

[8] Abd El-Karim S.S., Anwar M.M., Syam Y.M., Nael M.A., Ali H.F., Motaleb M.A. (2018). Rational design and synthesis of new tetralin-sulfonamide derivatives as potent anti-diabetics and DPP-4 inhibitors: 2D & 3D QSAR, in vivo radiolabeling and bio distribution studies. Bioorg. Chem..

[9] Ferraroni M., Angeli A., Pinteala M., Supuran C.T. (2022). Sulfonamide diuretic azosemide as an efficient carbonic anhydrase inhibitor. J. Mol. Struct..

[10] Van Berkel M.A., Elefritz J.L. (2018). Evaluating off-label uses of acetazolamide. Am. J. Health-Sys. Pharm..

[11] Košak U., Brus B., Knez D., Žakelj S., Trontelj J., Pišlar A., Šink R., Jukič M., Živin M., Podkowa A. (2018). The Magic of Crystal Structure-Based Inhibitor Optimization: Development of a Butyrylcholinesterase Inhibitor with Picomolar Affinity and in Vivo Activity. J. Med. Chem..

[12] Shetnev A., Shlenev R., Efimova J., Ivanovskii S., Tarasov A., Petzer A., Petzer J.P. (2019). 1,3,4-Oxadiazol-2-ylbenzenesulfonamides as privileged structures for the inhibition of monoamine oxidase B. Bioorg. Med. Chem. Lett..

[13] Abdel-Aziz A.A., Angeli M.A., El-Azab A.S., Hammouda M.E.A., El-Sherbeny M.A., Supuran C.T. (2019). Synthesis and antiinflammatory activity of sulfonamides and carboxylates incorporating trimellitimides: Dual cyclooxygenase/carbonic anhydrase inhibitory actions. Bioorg. Chem..

[14] Wan Y., Fang G., Chen H., Deng X., Tang Z. (2021). Sulfonamide derivatives as potential anti-cancer agents and their SARs elucidation. Eur. J. Med. Chem..

[15] Dolensky J., Hinteregger C., Leitner A., Seebacher W., Saf R., Belaj F., Mäser P., Kaiser M., Weis R. (2022). Antiprotozoal Activity of Azabicyclo-Nonanes Linked to Tetrazole or Sulfonamide Cores. Molecules.

[16] Khan F., Mushtaq S., Naz S., Farooq U., Zaidi A., Bukhari S., Rauf A., Mubarak M. (2018). Sulfonamides as potential bioactive scaffolds. Curr. Org. Chem..

[17] Delijewski M., Haneczok J. (2021). AI drug discovery screening for COVID-19 reveals zafirlukast as a repurposing candidate. Med. Drug Discov..

[18] Jiang D., Chen J., Zan N., Li C., Hu D., Song B. (2021). Discovery of Novel Chromone Derivatives Containing a Sulfonamide Moiety as Anti-ToCV Agents through the Tomato Chlorosis Virus Coat Protein-Oriented Screening Method. J. Agric. Food Chem..

[19] White K., Esparza M., Liang J., Bhat P., Naidoo J., McGovern B.L., Williams M.A.P., Alabi B.R., Shay J., Niederstrasser H. (2021). Aryl Sulfonamide Inhibits Entry and Replication of Diverse Influenza Viruses via the Hemagglutinin Protein. J. Med. Chem..

[20] Selvakumar B., Gujjar N., Subbiah M., Elango K.P. (2018). Synthesis and antiviral study of 4-(7,7-dimethyl-4-(piperazin-1-yl)-5,6,7,8tetrahydroquinazolin-2-yl) morpholine derivatives. Med. Chem. Res..

[21] Kornii Y., Shablykin O., Shablykina O., Brovarets V. (2021). New 4-iminohydantoin sulfamide derivatives with antiviral and anticancer activity. Ukr. Bioorg. Acta..

[22] Shin Y.S., Lee J.Y., Noh S., Kwak Y., Jeon S., Kwon S., Jin Y.-h., Jang M.S., Kim S., Song J.H. (2021). Discovery of cyclic sulfonamide derivatives as potent inhibitors of SARS-CoV-2. Bioorg. Med. Chem. Lett..

[23] Bendi A., Sangeeta S., Naina A. (2021). Study on Camphor Derivatives and Its Applications: A Review. Curr. Org. Chem..

[24] Sokolova A.S., Baranova D.V., Yarovaya O.I., Baev D.S., Polezhaeva O.A., Zybkina A.V., Shcherbakov D.N., Tolstikova T.G., Salakhutdinov N.F. (2019). Synthesis of (1*S*)-(+)-camphor-10-sulfonic acid derivatives and investigations in vitro and in silico of their antiviral activity as the inhibitors of fi lovirus infections. Russ. Chem. Bull..

[25] Kurul F., Doruk B., Topkaya S.N. (2025). Principles of green chemistry: Building a sustainable future. Discov. Chem..

[26] Eid N., Karamé I., Andrioletti B. (2018). Straightforward and sustainable synthesis of sulfonamides in water under mild conditions. Eur. J. Org. Chem..

[27] Qadir M.A., Ahmed M., Iqbal M. (2015). Synthesis, characterization, and antibacterial activities of novel sulfonamides derived through condensation of amino group containing drugs, amino acids, and their analogs. BioMed Res. Int..

[28] Xu J. (2021). Synthesis of Sulfonopeptides. J. Pept. Sci..

[29] Lakrout S., K’tir H., Amira A., Berredjem M., Aouf N.-E. (2014). A simple and eco-sustainable method for the sulfonylation of amines under microwave-assisted solvent-free conditions. RSC Adv..

[30] Liu W., Chen J., Su W. (2024). Recent advances in the synthesis of sulfonamide intermediates. Pharm. Front..

[31] Breitmaier E. (2006). Terpenes: Flavors, Fragrances, Pharmaca, Pheromones.

[32] Shokova E.A., Kim J.K., Kovalev V.V. (2016). Camphor and its derivatives. Unusual transformations and biological activity. Russ. J. Org. Chem..

[33] Vollmer B., Ebel H., Rees R., Nentwig J., Mulvaney T., Schünemann J., Krull J., Topf M., Görlich D., Grünewald K. (2025). A nanobody specific to prefusion glycoprotein B neutralizes HSV-1 and HSV-2. Nature.

[34] Wang C., Hesketh E.L., Shamorkina T.M., Li W., Franken P.J., Drabek D., van Haperen R., Townend S., van Kuppeveld F.J.M., Grosveld F. (2022). Antigenic structure of the human coronavirus OC43 spike reveals exposed and occluded neutralizing epitopes. Nat. Commun..

[35] Conley M.J., McElwee M., Azmi L., Gabrielsen M., Byron O., Goodfellow I.G., Bhella D. (2019). Calicivirus VP2 forms a portal-like assembly following receptor engagement. Nature.

[36] Petkova Z., Vilhelmova-Ilieva N., Dikova K. (2025). Antiviral Evaluation of Camphor-Based Sulfonamides against Structurally Diverse Viruses. Acta Microbiol. Bulg..

[37] Reed L.J., Muench H. (1938). A simple method of estimating fifty percent endpoints. Am. J. Hyg..

[38] Borenfreund E., Puerner J.A. (1985). Toxicity determination in vitro by morphological alterations and neutral red absorption. Toxicol. Lett..

[39] (2025). Cytopathic Effect Inhibition Assay. Creative Diagnostics. https://antiviral.creative-diagnostics.com/cc50-ic50-assay.html.

[40] Sewall L.M., de Paiva Froes Rocha R., Gibson G., Louie M., Xie Z., Bangaru S., Tran A.S., Ozorowski G., Mohanty S., Beutler N. (2025). Microfluidics combined with electron microscopy for rapid and high-throughput mapping of antibody-viral glycoprotein complexes. Nat. Biomed. Eng..

[41] Yang Y., Yao K., Repasky M.P., Leswing K., Abel R., Shoichet B.K., Jerome S.V. (2021). Efficient exploration of chemical space with docking and deep learning. J. Chem. Theory Comput..

