# Supplementary material for: Camphor-10-Sulfonamide Amino Acid Esters: Synthesis, Antiviral Evaluation, and Molecular Docking Insights
Source: Int J Mol Sci. 2026 Jan 7;27(2):616. doi: 10.3390/ijms27020616 (PMC12841417; doi:10.3390/ijms27020616)
Supplement: Supplementary file 1 [file ijms-27-00616-s001.zip › ijms-4011071-supplementary.pdf]

Supporting Information

Camphorsulfonamide amino acid esters: synthesis, antiviral evaluation, and molecular docking insights

Krasimira Dikova[1,2], Neli Vilhelmova-Ilieva[3*], Emilio Mateev[4], Zhanina Petkova[1,2*]

[1]Laboratory Organic Synthesis and Stereochemistry, Institute of Organic Chemistry with Centre of Phytochemistry, Bulgarian Academy of Sciences, Sofia, Bulgaria
[2]Centre of Competence "Sustainable Utilization of Bio-resources and Waste of Medicinal and Aromatic Plants for Innovative Bioactive Products" (BIORESOURCES BG), Sofia 1000, Bulgaria
[3]The Stephan Angeloff Institute of Microbiology, Bulgarian Academy of Sciences, Acad. G. Bonchev str., 26, Sofia 1113, Bulgaria
[43]Department of Pharmaceutical chemistry, Faculty of Pharmacy, Medical University, Sofia, Bulgaria

* Correspondence: nelivili@gmail.com; zhanina.petkova@orgchm.bas.bg

Table of content

**Content**	**Page**
NMR spectra of compound **5a**	2
NMR spectra of compound **5b**	4
NMR spectra of compound **6a**	6
NMR spectra of compound **6b**	8
NMR spectra of compound **7a**	10
NMR spectra of compound **7b**	12
HRMS spectra of compound **5a**	13
HRMS spectra compound **5b**	14
HRMS spectra of compound **6a**	14
HRMS spectra compound **6b**	15
HRMS spectra of compound **7a**	16
HRMS spectra compound **7b**	17
All active conformations on the tested compounds in the target proteins	19

Original NMR spectra

NMR spectra of compound **5a**

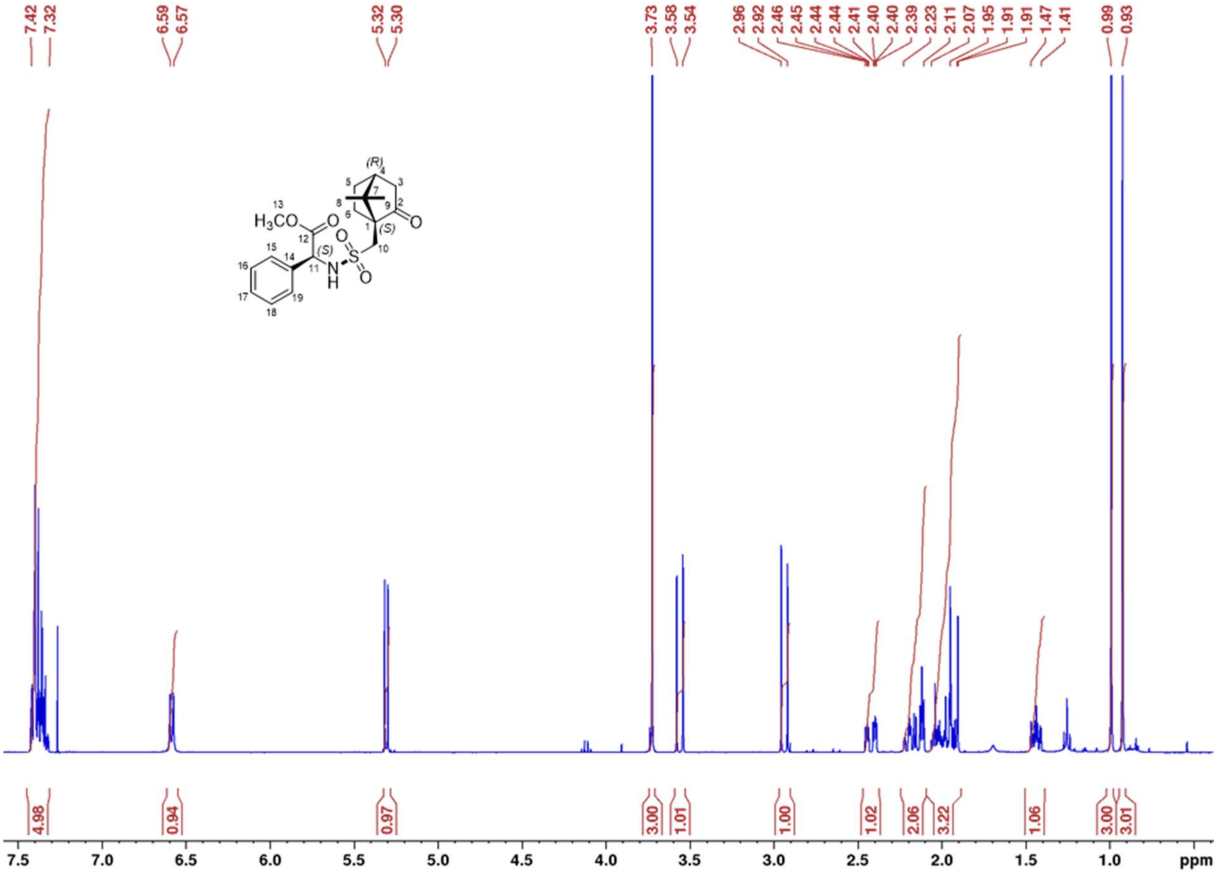

Figure S1. ^{1}H NMR spectrum of compound **5a** in $CDCl_3$.

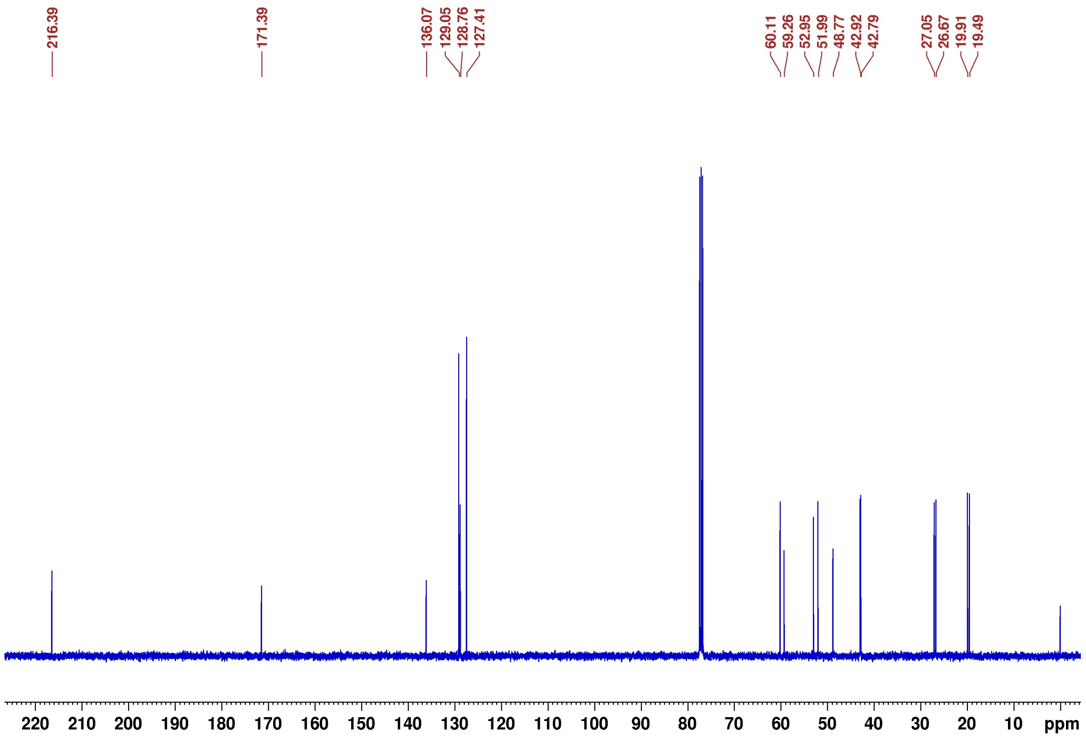

Figure S2. ^{13}C NMR spectrum of compound **5a** in $CDCl_3$.

NMR spectra of compound **5b**

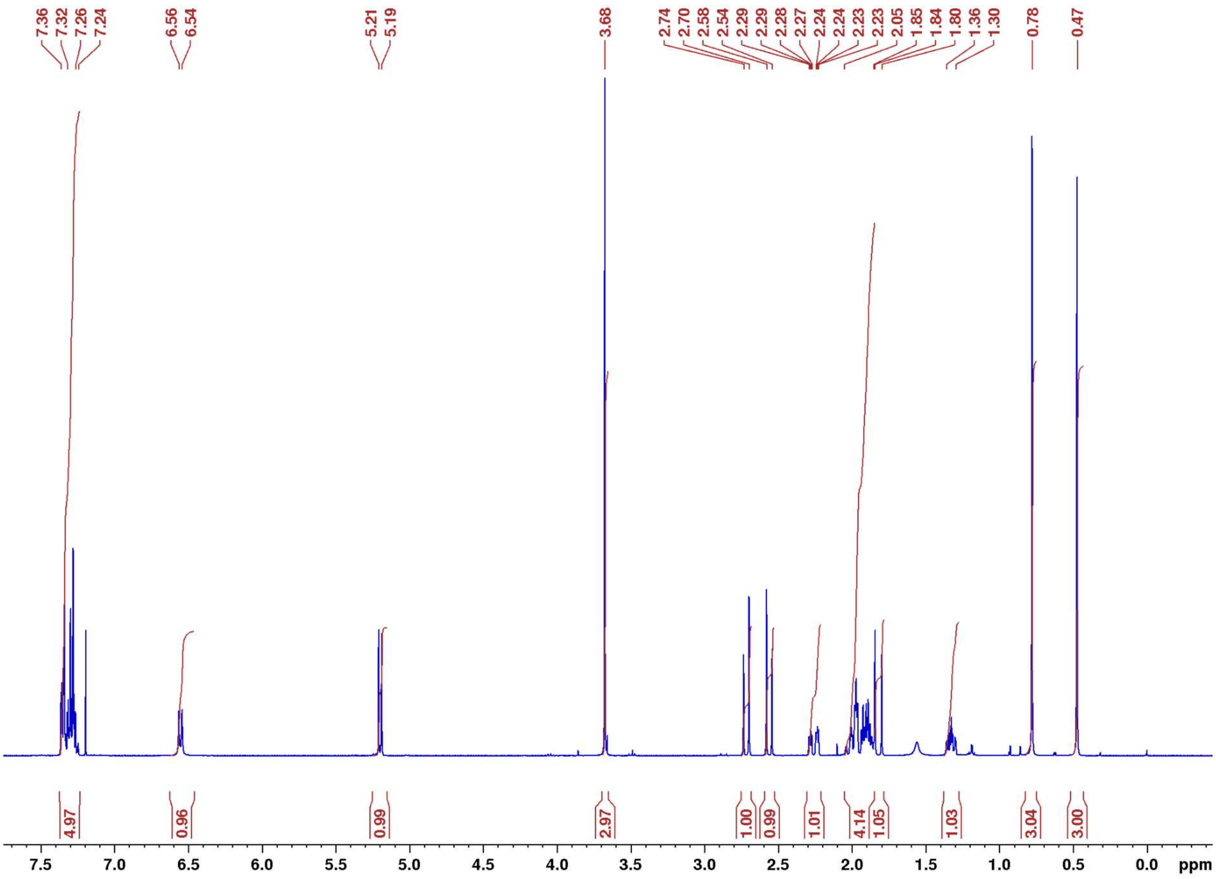

Figure S3. ^{1}H NMR spectrum of compound **5b** in $CDCl_3$.

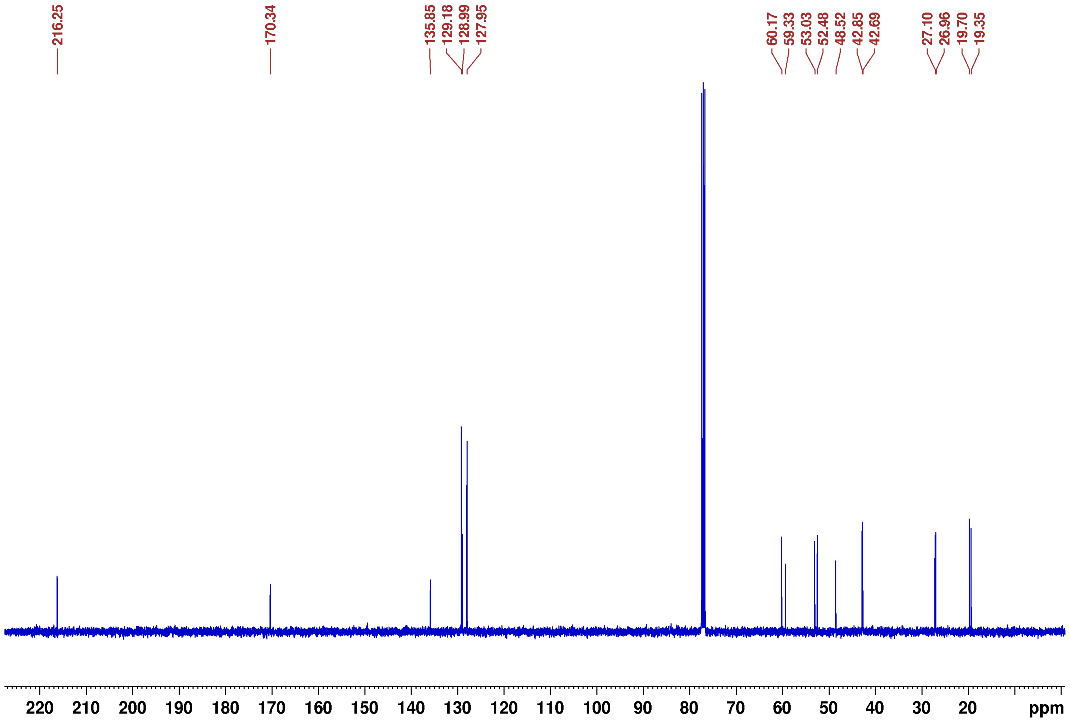

Figure S4. ^{13}C NMR spectrum of compound **5b** in $CDCl_3$.

NMR spectra of compound **6a**

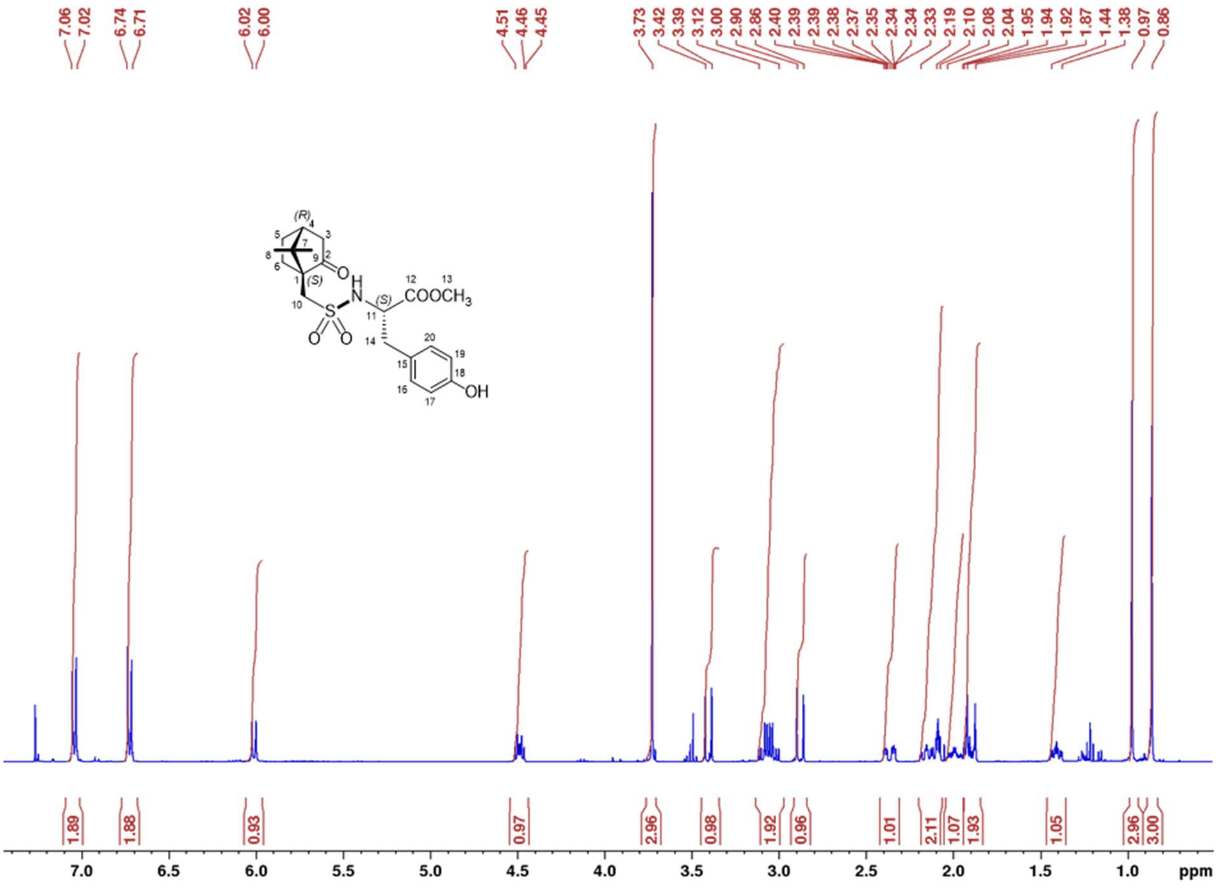

Figure S5. ^{1}H NMR spectrum of compound **6a** in $CDCl_3$.

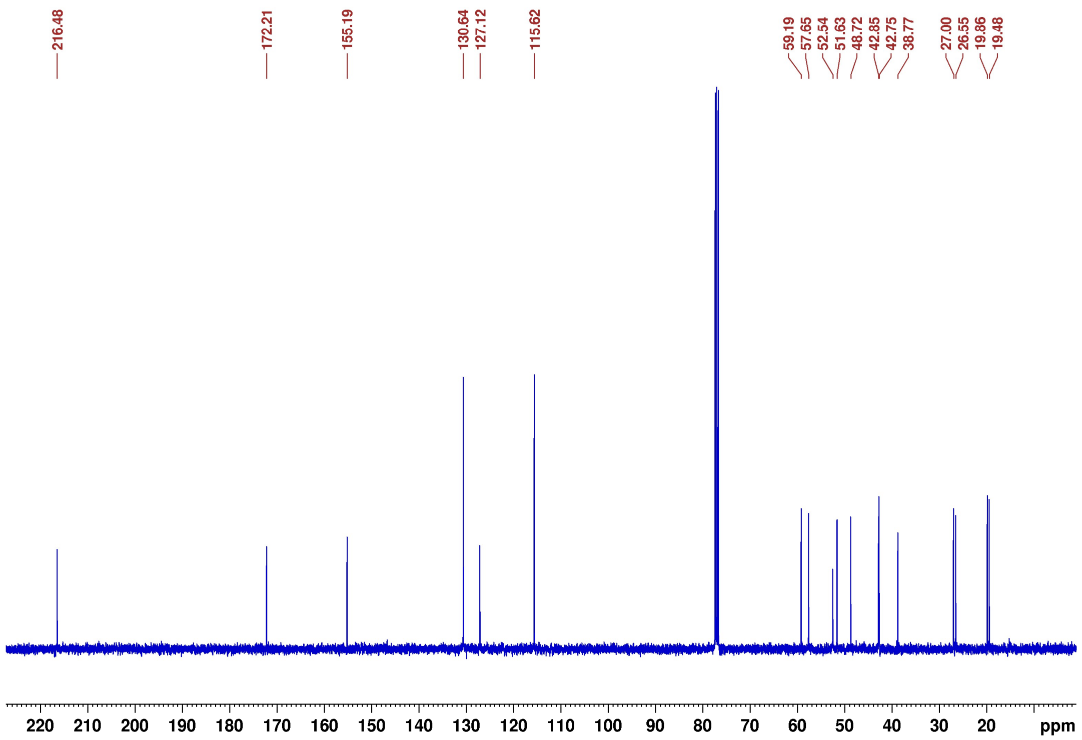

Figure S6. ^{13}C NMR spectrum of compound **6a** in $CDCl_3$.

NMR spectra of compound **6b**

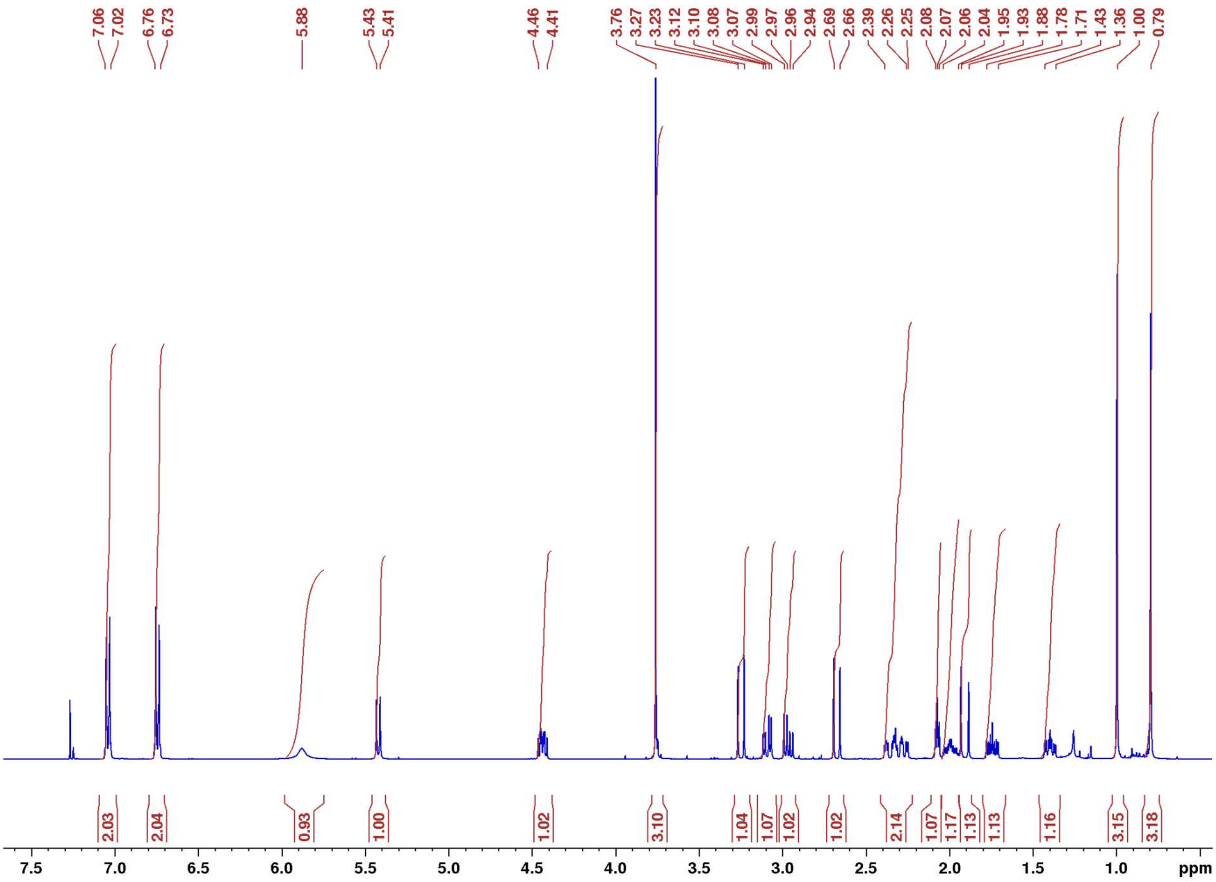

Figure S7. ^{1}H NMR spectrum of compound **6b** in $CDCl_3$.

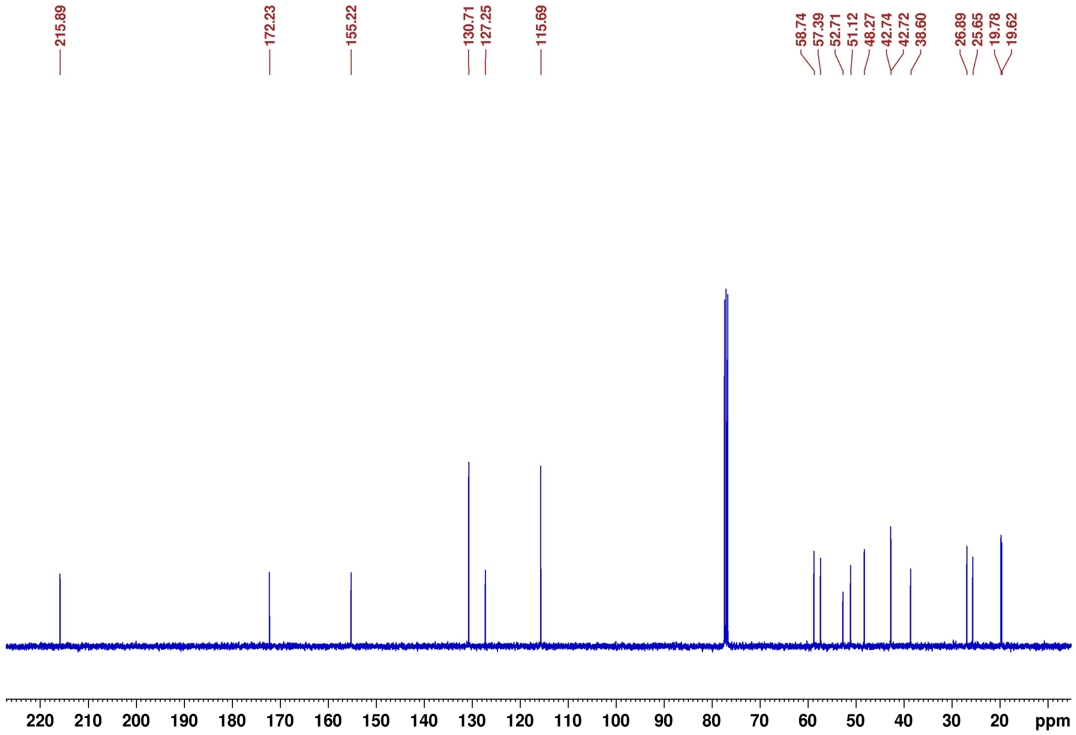

Figure S8. ^{13}C NMR spectrum of compound **6b** in $CDCl_3$.

NMR spectra of compound **7a**

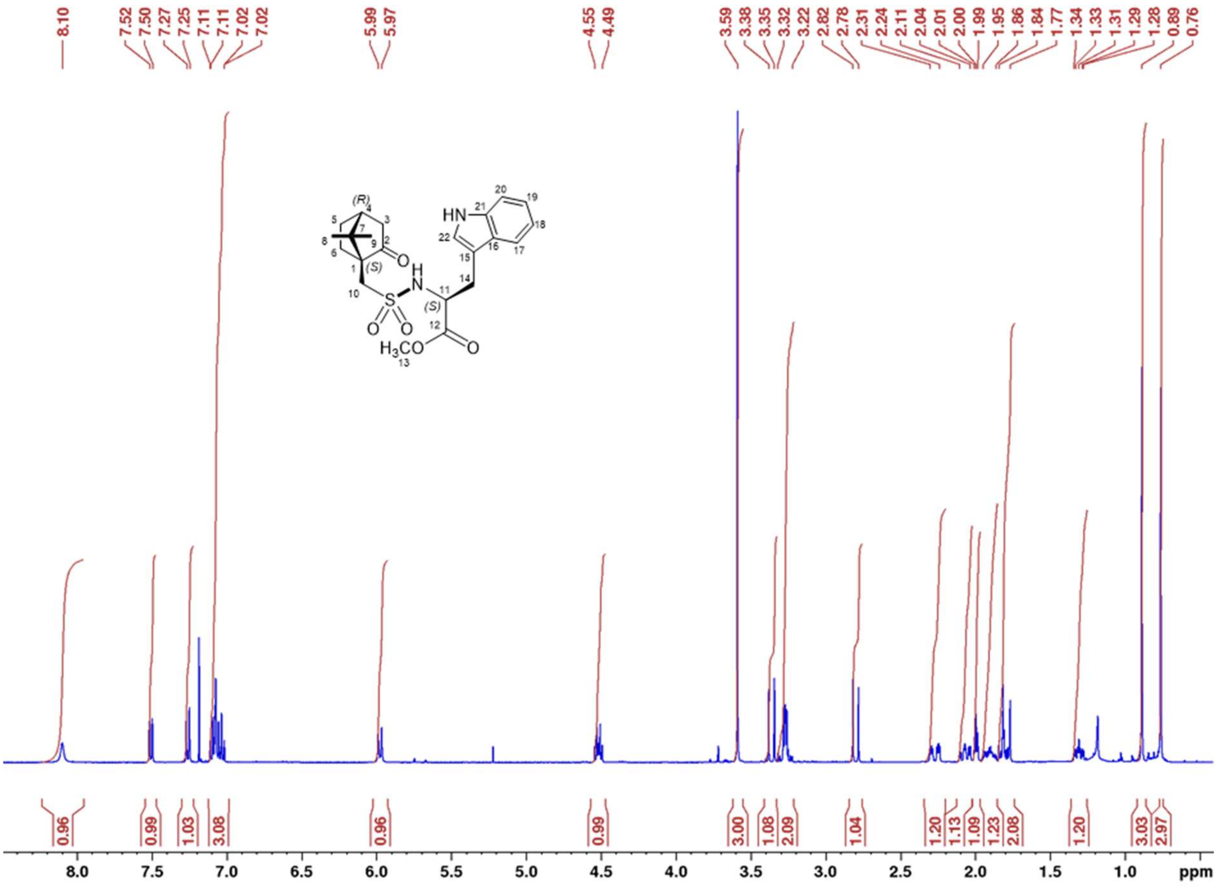

Figure S9. ^{1}H NMR spectrum of compound **7a** in $CDCl_3$.

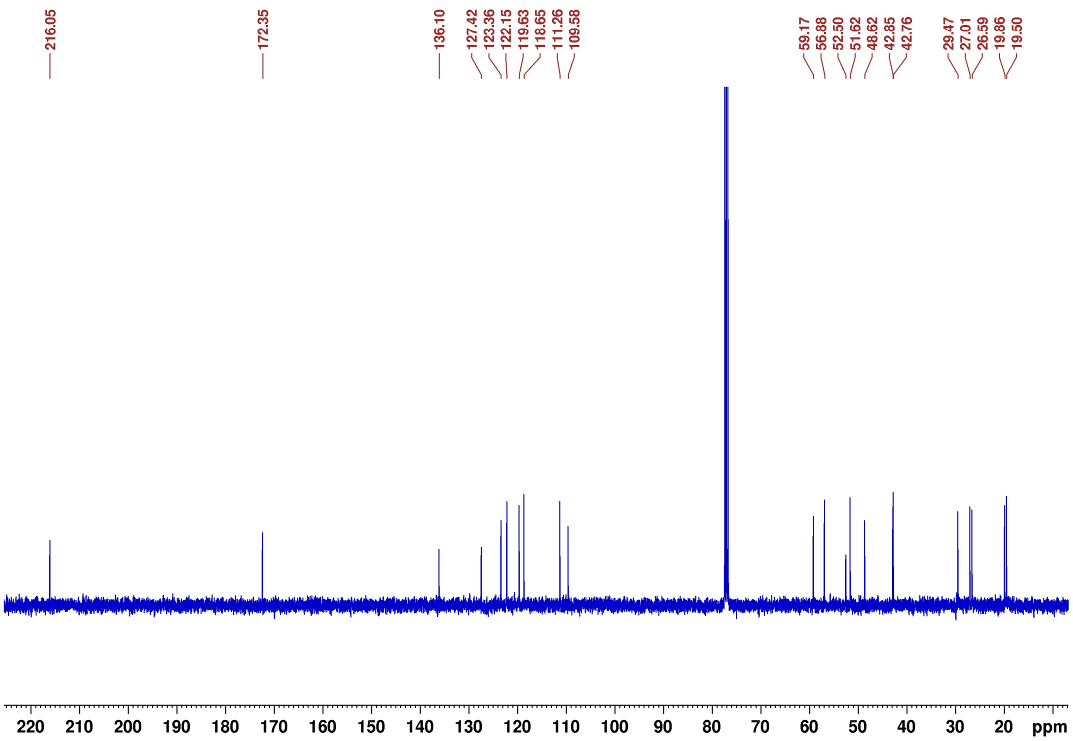

Figure S10. ^{13}C NMR spectrum of compound **7a** in $CDCl_3$.

NMR spectra of compound **7b**

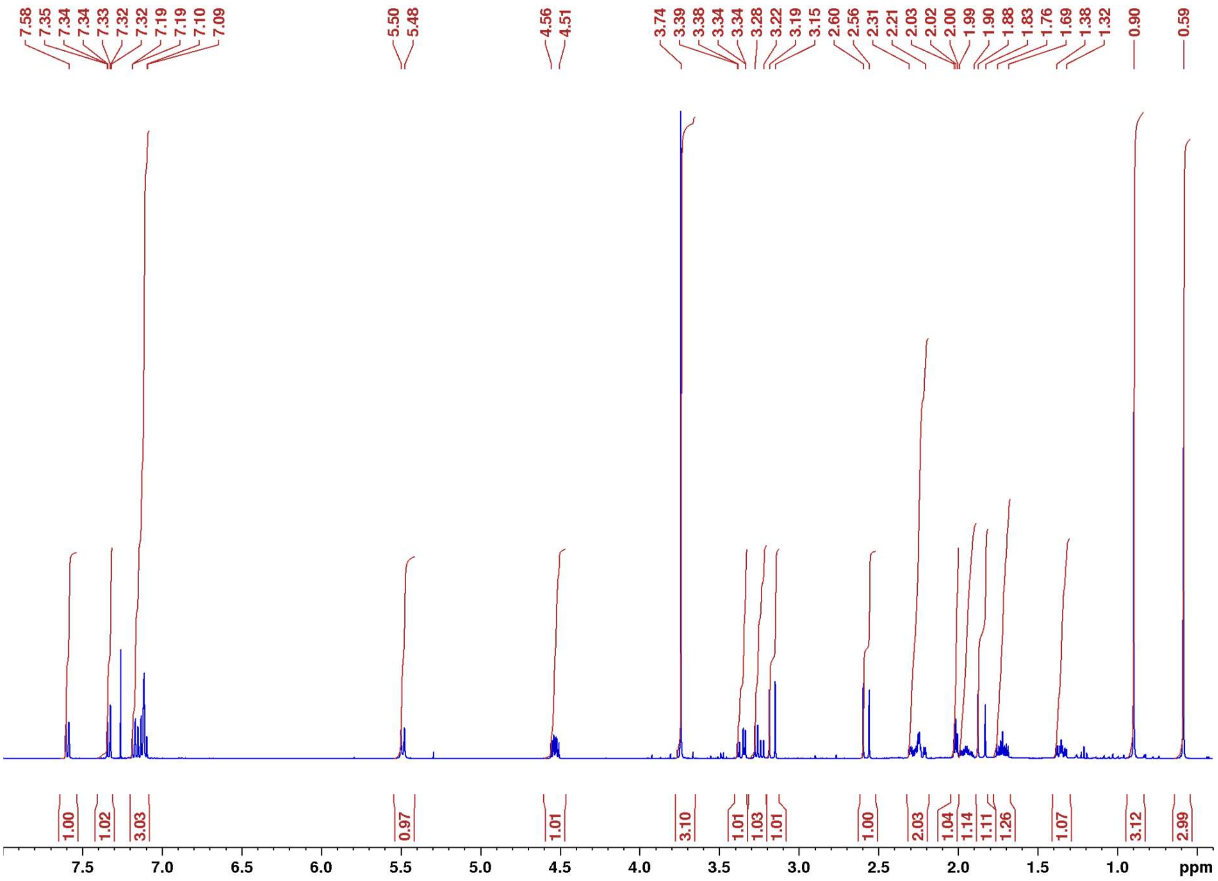

Figure S11. ^{1}H NMR spectrum of compound **7b** in $CDCl_3$.

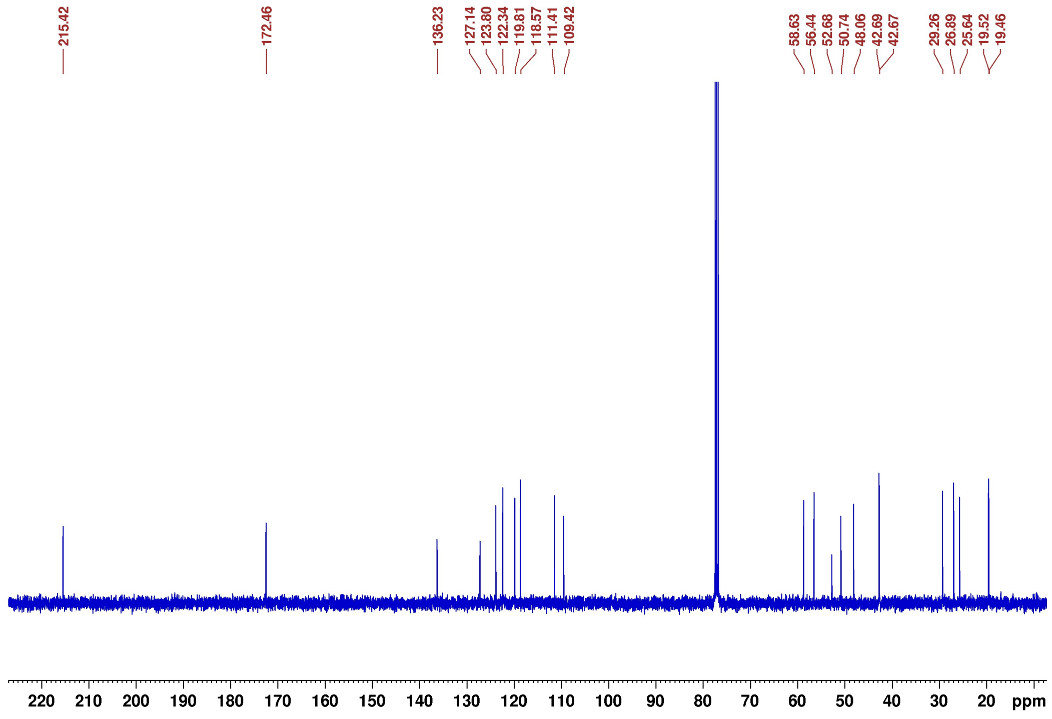

Figure S12. ^{13}C NMR spectrum of compound **7b** in $CDCl_3$.

Original HESI HR-MS spectra

<u>HRMS spectra of compound **5a**</u>

Figure S13. HRMS ($HESI^+$) spectrum of compound **5a**.

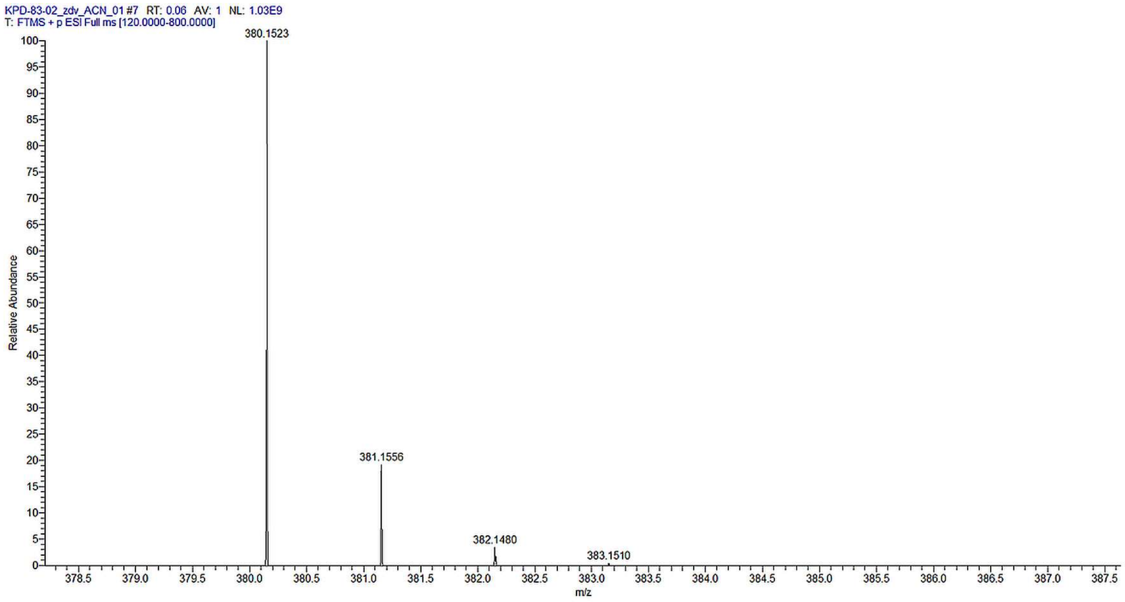

Figure S14. HRMS ($HESI^-$) spectrum of compound **5a**.

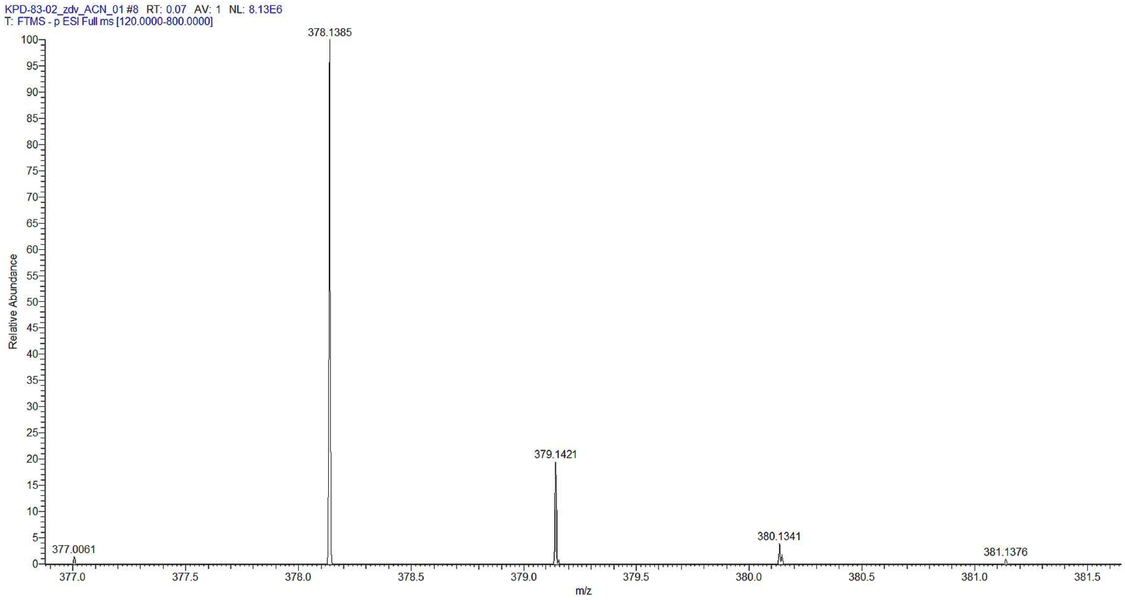

HRMS spectra of compound **5b**

Figure S15. HRMS (HESI$^+$) spectrum of compound **5b**.

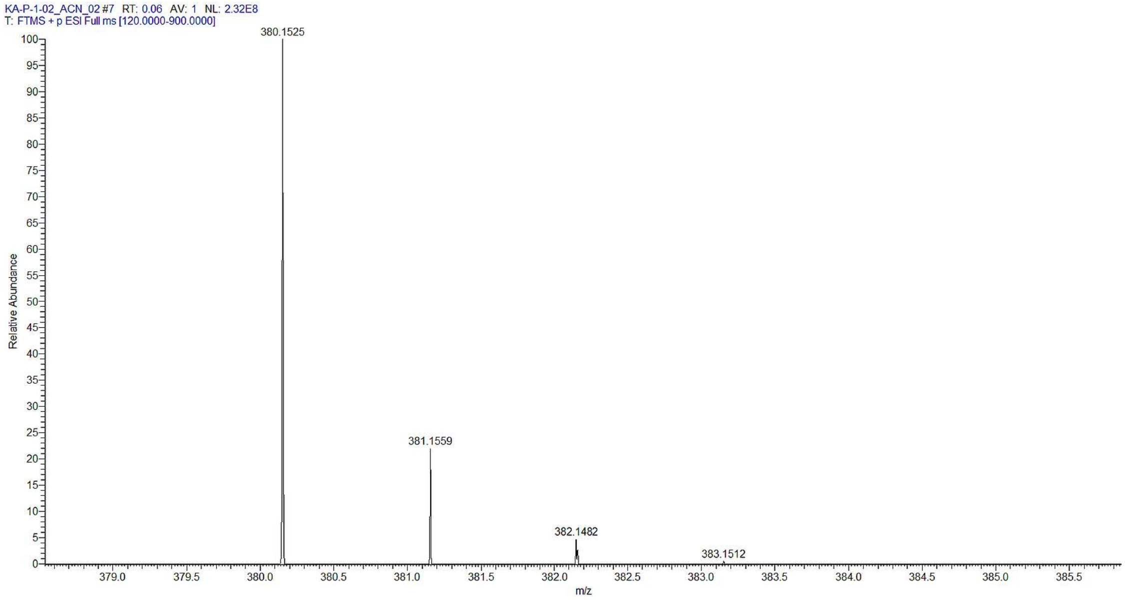

HRMS spectra of compound **6a**

Figure S16. HRMS (HESI$^+$) spectrum of compound **6a**.

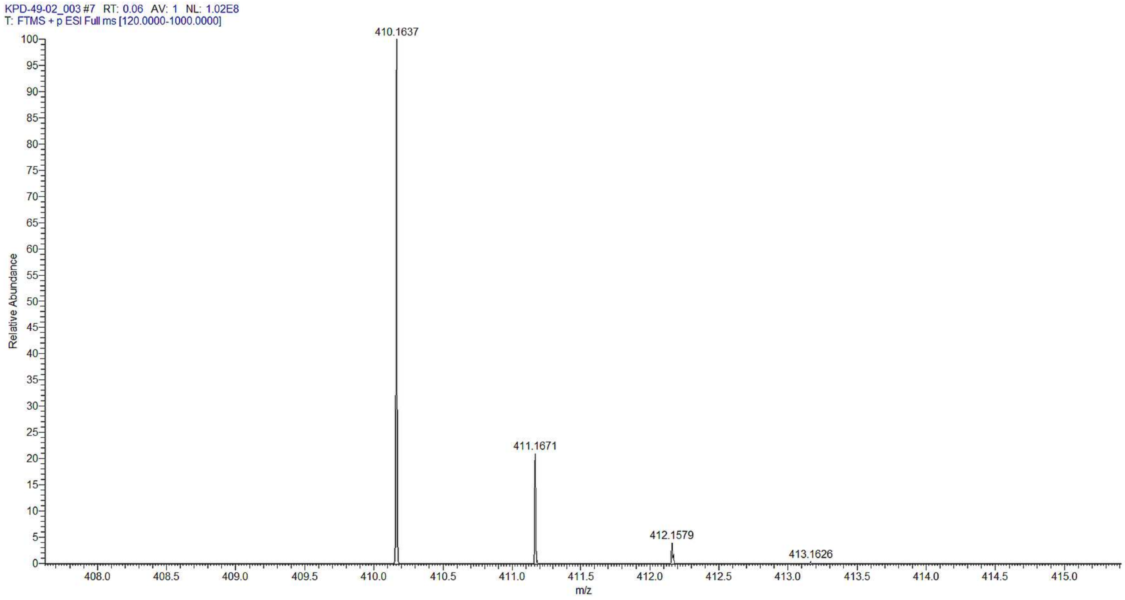

Figure S17. HRMS (HESI$^-$) spectrum of compound **6a**.

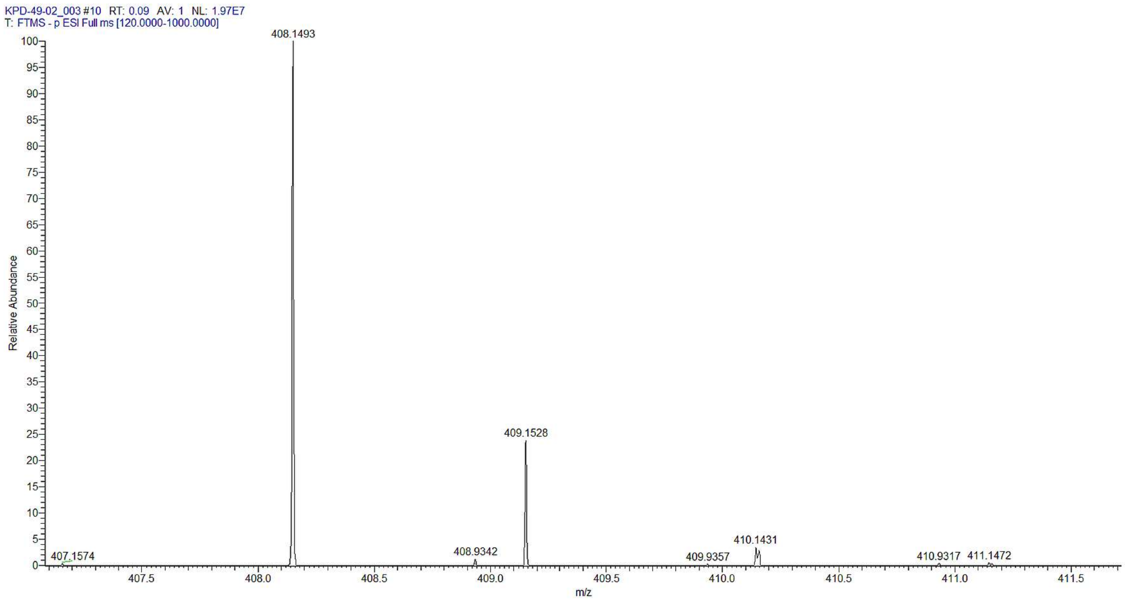

<u>HRMS spectra of compound **6b**</u>

Figure S18. HRMS (HESI$^+$) spectrum of compound **6b**.

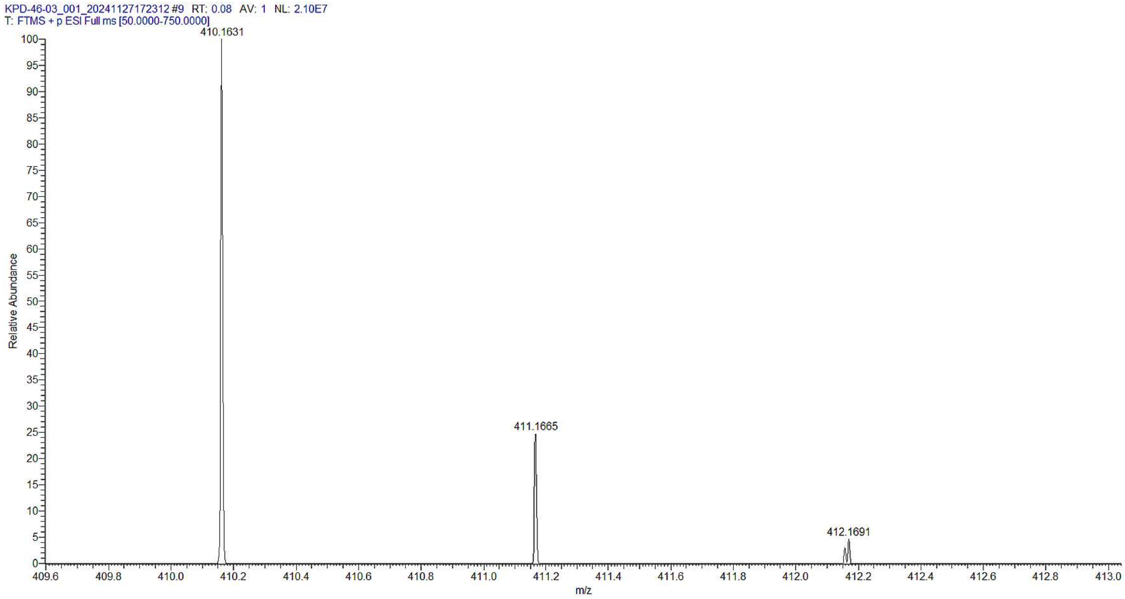

Figure S19. HRMS (HESI$^-$) spectrum of compound **6b**.

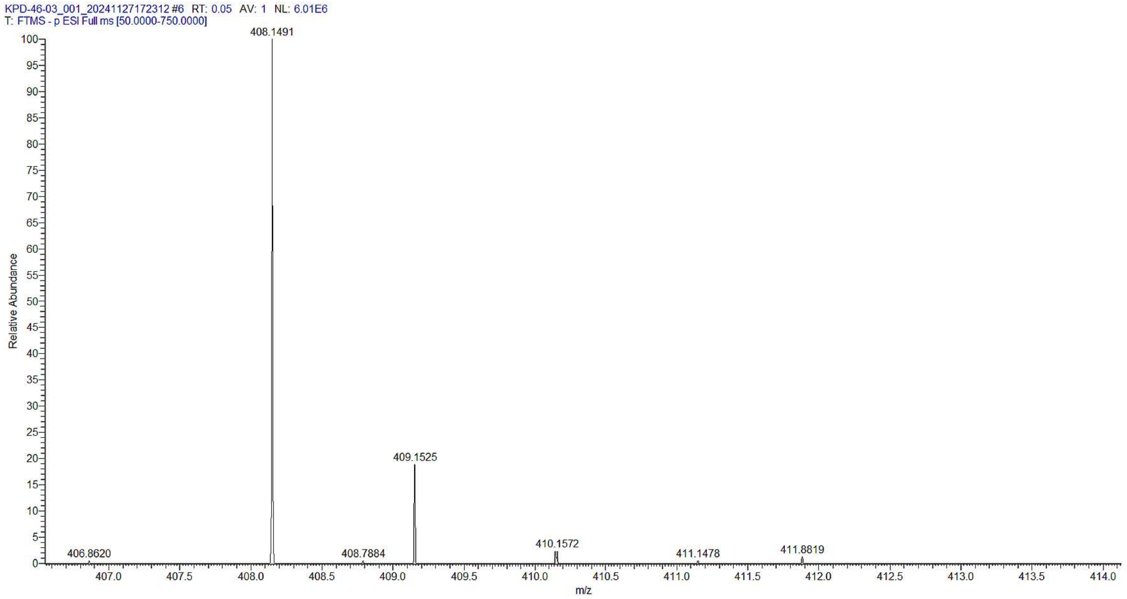

HRMS spectra of compound **7a**

Figure S20. HRMS (HESI$^+$) spectrum of compound **7a**.

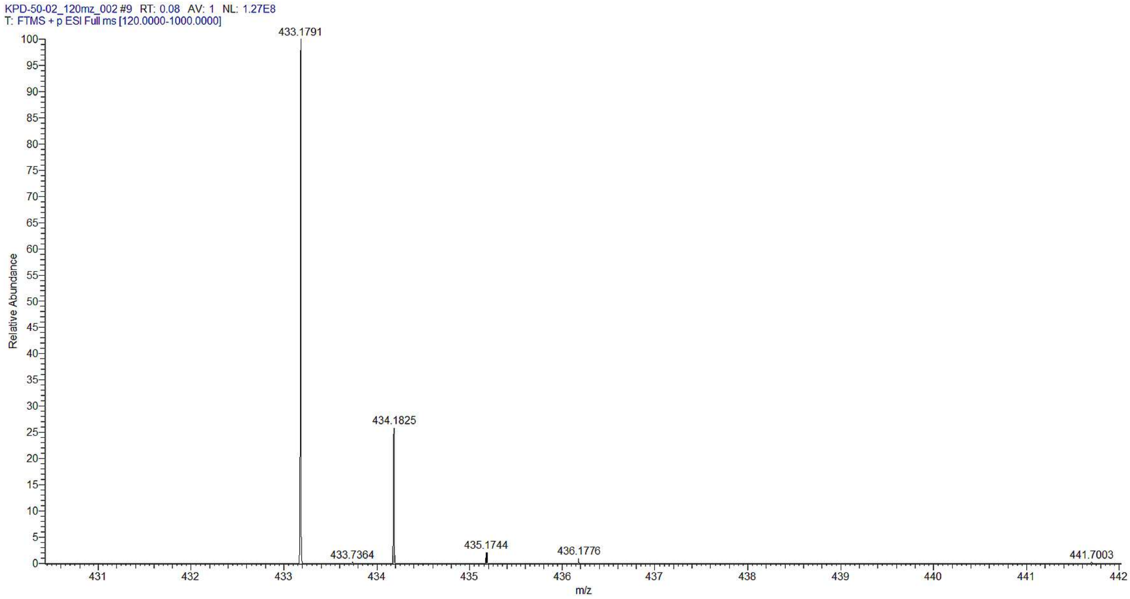

Figure S21. HRMS (HESI$^-$) spectrum of compound **7a**.

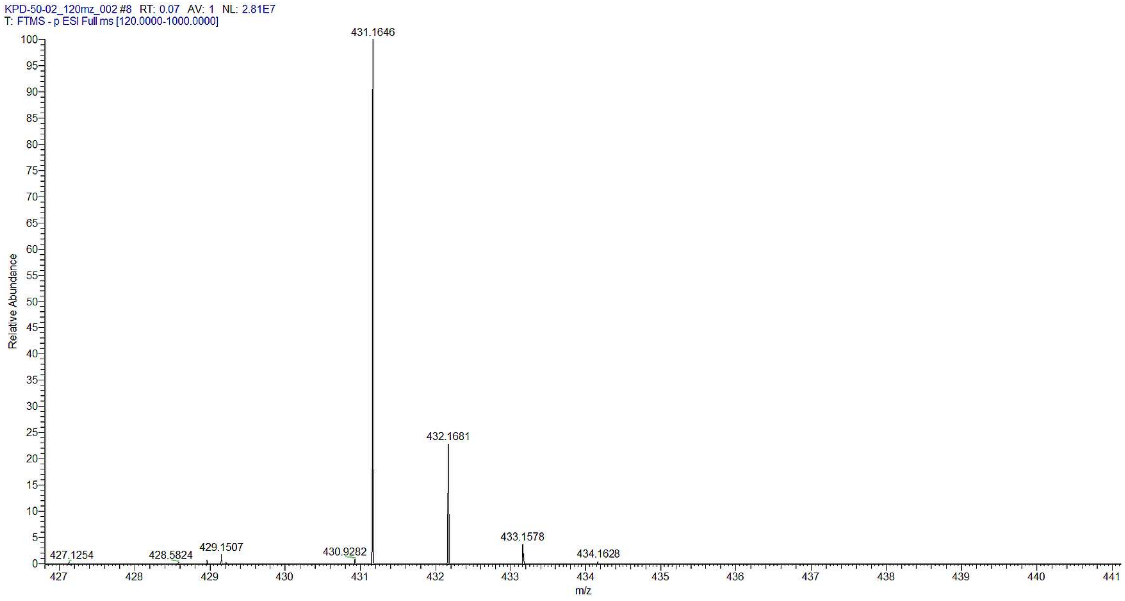

HRMS spectra of compound **7b**

Figure S22. HRMS (HESI$^+$) spectrum of compound **7b**.

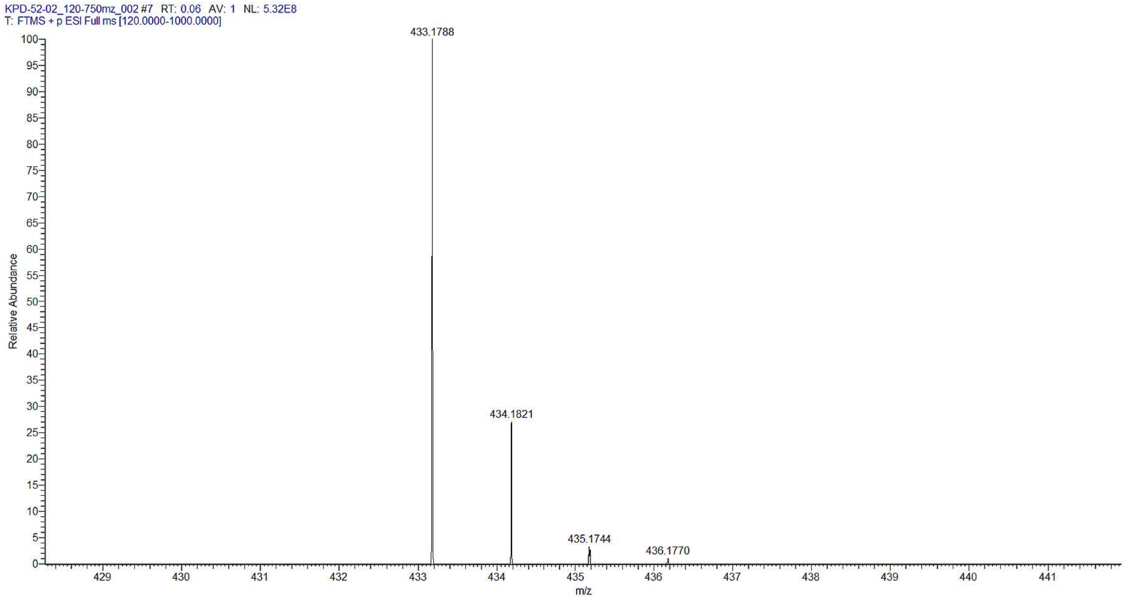

Figure S23. HRMS (HESI$^-$) spectrum of compound **7b**.

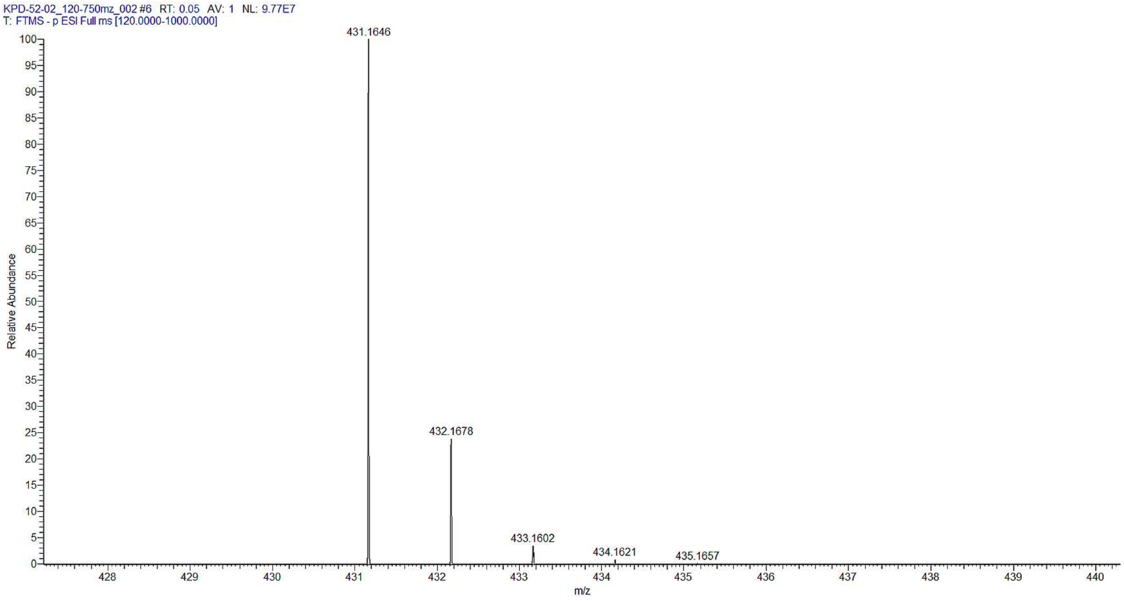

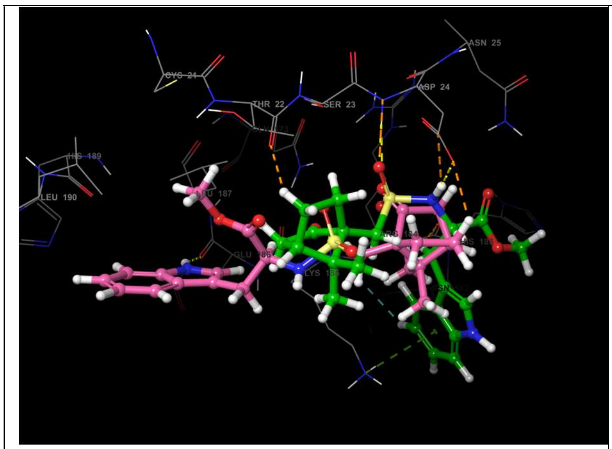	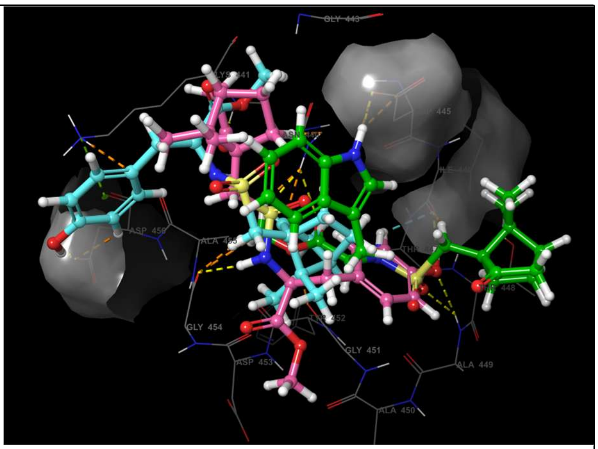
**A**	**B**
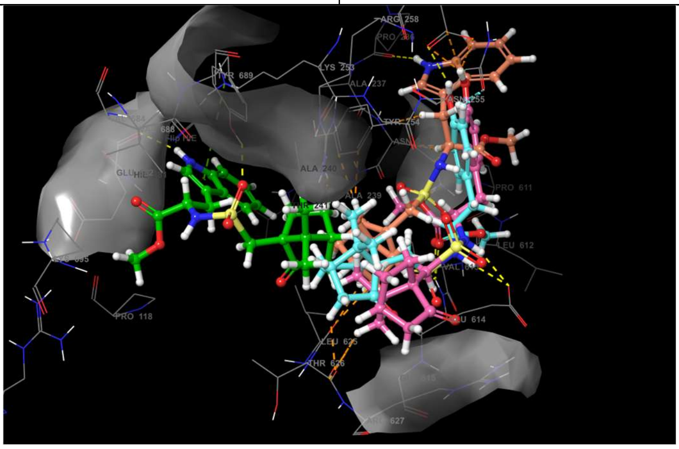	
**PC**	

Figure S24. Top-ranked docking poses of the tested camphor-based compounds in the target viral proteins. Panel A: Binding conformations in HCoV-OC43 spike protein (PDB: 9BLK). Panel B: Binding conformations in FCV capsid P-domain protein (PDB: 6GSH). Panel C: Binding conformations in HSV-1 glycoprotein (PDB: 9Q9L).
